# Expert consensus for a national essential antidote list: E-Delphi method

**DOI:** 10.1371/journal.pone.0269456

**Published:** 2022-06-16

**Authors:** Dalal Al-Taweel, Samuel Koshy, Sara Al-Ansari, Asmaa Al-Haqan, Bedoor Qabazard

**Affiliations:** 1 Department of Pharmacy Practice, College of Pharmacy, Kuwait University, Jabriya, Kuwait; 2 Pharmacist, Ministry of Health, Kuwait, Kuwait; 3 Department of Pharmacology and Therapeutics, College of Pharmacy, Kuwait University, Jabriya, Kuwait; ADRZ: Admiraal De Ruyter Ziekenhuis, NETHERLANDS

## Abstract

Antidote stocking represents a major challenge to hospitals all over the world, including Kuwait. In order to assist hospitals to reduce costs and improve patient care, an essential antidote list can be used as an initial foundation for securing sufficient antidote availability at healthcare institutions. The aim of our study is to generate a nationally relevant essential antidote list for emergency care hospitals in Kuwait using the e-Delphi method by establishing consensus through a multidisciplinary expert group of healthcare providers. An electronic survey with 47 essential antidotes was developed. The e-Delphi method was used, with three rounds of voting, to determine expert consensus on an essential antidote list for hospitals in Kuwait. A purposive sample of healthcare professionals from governmental and private hospitals were selected for this study (n = 30). Consensus was gained if ≥75% of the expert panel agreed on the inclusion of the antidote, without any strong disagreements. Round 1 of the e-Delphi resulted in 41 antidotes reaching consensus and seven new antidotes suggested by the expert panel. Round 2 had two antidotes (out of seven newly suggested ones) reaching consensus. Round 3 was a confirmatory round, where the expert group agreed on their previous rounds’ opinions. This resulted in the development of an essential antidote list with 43 antidotes. The optimal approach for ensuring adequate availability of antidotes is continuous monitoring of local poisoning incidence and antidote requirements through collaborations between academic researchers and emergency care clinicians. The development of an essential antidote list, with expert consensus, is one of the initial steps in securing a foundation for appropriate provision of antidotes at all healthcare institutions. This is the first study that the authors are aware of that demonstrates that the e-Delphi technique can consolidate recommendations of experts in emergency medicine to provide a list of essential antidotes.

## Introduction

Poisoning is a paramount public health issue that is responsible for frequent visits to the emergency department and hospital admissions. Effective management of poisoning requires adequate availability of antidotes, with supportive measures, in hospitals that provide effective emergency care [1, 2]. Antidotes have a critical role in the emergency care of a patient admitted for poisoning or drug overdose, as it prevents death, and decreases the duration of therapy and hospital stay. Studies have reported inadequate availability of antidotes in emergency care facilities, which experts warn will lead to increased morbidity and mortality [1, 3–5]. Unfortunately, the stocking of antidotes has been a “persistent concern” for over 35 years [1]. Countries have conducted regular audits on antidotes stocking, and many researchers have recommended the development of a national list and stocking guidelines to standardize care [4, 6, 7]. A globally recognized consensus guideline recommended a “list” of antidotes to be stocked for timely delivery of essential antidotes to the emergency department [1]. It is advocated that this list must be modified and adopted by each healthcare institute based on a hazard vulnerability assessment (HVA) to meet local antidote needs [1, 8]. It is also highly recommended that the list should be determined by the concerned stakeholders consisting of physicians, pharmacists, and other healthcare professionals associated with emergency care [1, 9]. Organizing a meticulous HVA is challenging as it is resource-intensive and time-consuming, requiring comprehensive face-to-face interactions. It requires collaboration between hospitals and other institutions as each hospital is challenged with its own specific geographic, political and social features. In the absence of a rigorous HVA, the list of essential antidotes can be created by reaching consensus within expert groups, by employing methods such as the Delphi technique, the nominal group technique or through a consensus development conference [10].

The Delphi method was developed in the 1950s and became extensively accepted for gathering and merging opinions on a particular topic to set goals and investigate policies. It is well adapted for building consensus, with a succession of questionnaires, to procure data from a group of selected members [11]. The Delphi method has been used to establish consensus in emergency medicine, including antidote stocking [12–14]. The e-Delphi method is a computerized version of the Delphi method that depends on an internet-based platform offering tremendous savings in time and cost, and advantages in data management [15].

In Kuwait, a country with a population of 4.3 million [16], approximately 1150 cases of poisonings due to medications and chemicals are reported annually [17]. A prospective study in Kuwait indicated an increasing prevalence of poisoning cases, with 57.8% of patients admitted for intentional poisoning, and 42.2% for unintentional poisoning [17]. Drug overdose toxicity is the third most common cause of accidental deaths in the country [18]. A 2010 study in Kuwait reported that approximately 35% to 50% of poisoning cases between 2000 and 2005 were pediatric poisonings due to drugs and biological substances [19]. A 2020 national audit of antidote stocking in Kuwait concluded that there is inadequate stocking of some antidotes in both the public and private hospital sector. The same study recommended the development of national guidelines for antidote stocking in Kuwait and the customization of guidelines to suit the local poisoning cases [8]. To assist hospitals to reduce costs and improve patient care, there is an urgent need to develop expert consensus guidelines, including a locally relevant list of essential antidotes, through a systematic, explicit, and transparent decision-making process [8].

The aim of our study is to generate a nationally relevant list of essential antidotes to secure adequate availability of antidotes in hospitals that provide emergency care in Kuwait using the e-Delphi technique. To our knowledge, this is the first study that uses an e-Delphi technique to consolidate recommendations of experts in emergency medicine to provide a list of essential antidotes for the management of adult patients with poisoning.

## Methods

### Study design and population

The e-Delphi method was used in this study, with three rounds of voting, to determine expert consensus on the development of an essential antidote list for hospitals in Kuwait that provide emergency care services. Ethics approval for this study was provided by the Health Science Centre Ethics Committee at Kuwait University as well as the Ministry of Health Ethics committee (Ethics number: 2019/1225).

A purposive sample of healthcare professionals from different geographical regions across Kuwait and with diverse specialties (including medical toxicology, emergency medicine, internal medicine, critical care medicine, pediatrics, pediatric critical care, hospital pharmacy and clinical pharmacy) from both governmental and private hospitals were selected for this study to form the expert panel. The additional inclusion criteria were that healthcare professionals were qualified as experts if they had i) at least 2 years’ post-qualification experience (unless they were an emergency medicine resident), ii) practiced in Kuwait for at least 2 years. Thirty experts were initially contacted and asked to participate in consensus development.

### Survey content and administration

A questionnaire was developed using the commercial survey software Qualtrics® (Provo, USA) with a preliminary list of 47 antidotes. This initial list was composed of antidotes retrieved from a national audit conducted in Kuwait in 2020 [8], that was based on evidence-based international guidelines [1, 20]. The research group (which included an academic toxicologist) reviewed in-depth the initial list and made necessary additions and deletions, as needed. Additionally, before commencing the Delphi process, the Delphi questionnaire was piloted with three emergency care physicians for their expert opinions. The purpose of this step of the process was to identify antidotes applicable for Kuwait and ensure that the Delphi process was evidence-based as well as consultative.

The survey instrument was composed of three sections. The first section collected the experts’ demographic data (e.g. site of practice, specialties, and position) in addition to their opinion on whether Kuwait needs to have its own list of essential antidotes and their knowledge on the existence of an essential antidote list. The second section included a list of 47 antidotes, with their relevant indication for use. Experts were asked to rate their level of agreement on whether to include each antidote in the essential antidote list for Kuwait on a four-point Likert scale (strongly agree/agree/disagree/strongly disagree). It has been demonstrated that four-point scales produce stable findings in Delphi studies [21]. In the third section, experts were asked to add any new antidotes that they considered to be needed in the essential list of antidotes in Kuwait that were not on our initial list. A comments box was added to allow experts to write their opinions.

Data were collected via an online questionnaire through three rounds between March-November 2020. The study survey was pre-tested on three experts; a specialist in emergency medicine, an internist and family medicine physician. They were asked to comment on the content, clarity and phrasing of the questions as well as the layout of the questionnaire. Their feedback informed the decision to include the indications for each antidote (as some antidotes are likely to have other indications not associated with emergency care).

A formal iterative process was conducted. The survey clearly displayed the objectives of the study and provided clear instructions for the experts on how to complete the survey. Experts were informed that they had the freedom to withdraw from the study at any time. As no consistent threshold for consensus exists [22], the following threshold was defined for our study: “Consensus was gained if ≥75% of the experts agreed on the inclusion of the antidote, provided there was no strong disagreement in the results”. Although the literature has reported many cut-offs for Delphi studies [23, 24], systematic reviews by Diamond et al. and Foth et al. have concluded that consensus is most commonly defined based on the percentage of agreement with a specific criterion, followed by the percentage of participants who rate items at the upper extremes of the Likert scales used (i.e. items scored agree or strongly agree on a Likert scale) [25, 26]. However, the range reported for consensus acceptance is very wide (50–97%) [27]. Experts were given two weeks to respond, with two reminders sent two weeks apart. The second and third questionnaire rounds were sent 4 weeks after the previous round was finished. To complete the e-Delphi process, experts were required to respond across all three rounds. Therefore, those who did not respond to Round 1 were not invited to participate in Round 2.

### e-Delphi rounds

Round 1 involved circulating the electronic survey to 30 experts. Experts indicated their level of agreement on the inclusion of each antidote in the essential list of antidotes for Kuwait and were also provided with the opportunity to add any new antidotes they believe should be included in the essential antidotes list. Experts were given two weeks to respond, with a reminder provided after one week. The results of Round 1 of the Delphi method were collated, and a summary of the responses was documented to provide a preliminary level of group consensus for each item.

A second questionnaire, showing the summary response of the whole group and the consensus level, was sent back to the experts in Round 2. The experts had the chance to reflect on their previous answers and re-rank their initial judgment in light of the group’s response. Any antidote that had not reached consensus or had gained a “strongly disagree” vote in Round 1, was re-rated. Additionally, any newly suggested antidotes from Round 1 were also voted on.

In Round 3, experts were provided with a summary response of the whole group for Round 2 and the consensus level. Experts were, again, given the opportunity to reflect on their answers for Round 2 and re-rank their initial judgment in light of the group’s response. Any antidote that had not reached consensus or had gained a “strongly disagree” vote in Round 2, was re-rated. Additionally, any newly suggested antidotes from Round 1 were also voted on.

### Patient and public involvement

Patients or the public were not involved in the design, or conduct, or reporting, or dissemination plans of our research.

### Data analysis

Data was exported from Qualtrics® to Microsoft Excel for analysis. Descriptive analysis was used to present the experts demographic characteristics as well as their levels of agreement for the inclusion of each individual antidote in numbers and percentages. Antidotes that were rated “strongly agree” or “agree” by at least 75% of the experts were included in the final list.

## Results

Of the 30 experts invited to participate in the study, 22 experts agreed to participate in our study. All 22 experts completed Round 1 and Round 2 (100% response rate), and 20 out of 22 experts completed Round 3 (90.9% response rate). Table 1 shows the demographics of the expert group in all three rounds. Gender distribution was consistent across all three rounds, with a higher percentage of males. More than half of the experts worked in the government sector, and specialized in emergency medicine. The majority of experts participating in the study were medical doctors, with only three pharmacists taking part.

**Table 1:** Demographics of experts in Rounds 1, 2 and 3.

Demographics	Round 1 (n = 22)	Round 2 (n = 22)	Round 3 (n = 20)
**Gender**			
Male	16	16	14
Female	6	6	6
**Site of practice** *			
Governmental	13	13	12
Private	8	8	7
Other	2	2	2
**Specialty** *			
Emergency Medicine	15	15	13
Pharmacy	3	3	3
Pediatrics	4	4	4
ICU	1	1	1
Medical Toxicology	1	1	1
**Position**			
Resident	1	1	1
Registrar	0	0	0
Assistant Registrar	0	0	0
Senior Registrar	7	7	5
Specialist	3	3	3
Senior Specialist	3	3	3
Consultant	5	5	5
Pharmacist	3	3	3

*Aggregates are more than the sample number as some experts worked in two sites/specialties.

When asked whether Kuwait needs to have its own list of essential antidotes, all experts, apart from one, agreed on Kuwait having its own essential antidote list. The participant who did not agree stated that in his opinion, “given the landscape, a more regional list may be more adequate”. Moreover, when experts were asked about whether they were aware of any essential antidote list currently used in Kuwait, the experts unanimously reported that they were not aware of any list or guidelines for antidote availability in Kuwait.

## e-Delphi rounds

**Fig 1:**
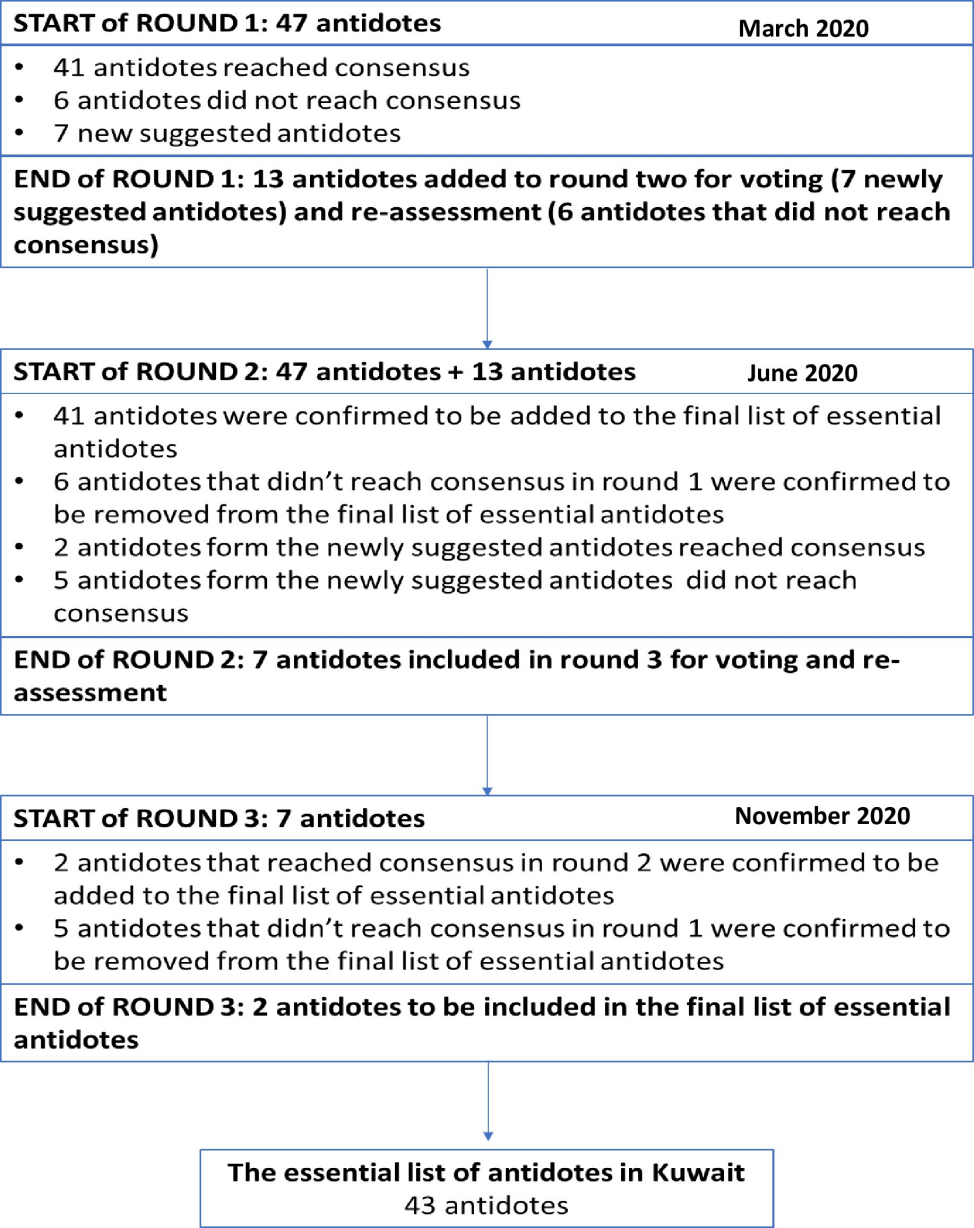
Step-by-step process of developing the expert consensus list of essential antidotes.

Fig 1 shows the step-by-step process of developing the expert consensus list of essential antidotes in Kuwait.

Details on each round are provided below:

Round one of the e-Delphi study asked the experts to rate their level of agreement to the inclusion of 47 antidotes in the essential antidotes list for Kuwait. In addition, experts were asked to suggest any essential antidote they believed should be added to the list. Overall, out of the 47 antidotes, the panel recommended 41 antidotes to be included in the essential antidote list; only 6 antidotes did not reach consensus (% consensus was <75% or had at least one strongly disagree answer), these were: acetylcysteine PO, dicobalt edetate, black widow spider antivenin, ethanol IV, mesna and Prussian blue. The six antidotes were re-assessed during Round 2. In addition, the experts suggested seven new antidotes to be added to the essential antidote list, these were: sugammadex, unithiol, glucarpidase, hyperbaric oxygen therapy, prothrombin complex concentrate, uridine triacetate and calcium trisodium. These were added to the questionnaire in round two.

Round two of the e-Delphi study consisted of two parts. The first part asked the experts to reflect on their ratings for those antidotes that did reach consensus in Round 1 (based on the group’s consensus level), and part two asked them to re-rate the 6 antidotes that did not reach consensus in Round 1 as well as the 7 newly suggested antidotes. All experts were happy with their Round 1 results for those antidotes that reached consensus (n = 41), and those were therefore added to the final list of essential antidotes in Kuwait (and were not included in Round 3). With regards to the 6 antidotes that did not reach consensus in Round 1, experts confirmed their responses and did not agree on having them added to the essential antidote list. The 6 antidotes were therefore removed and were not included in Round 3. With regards to the 7 newly suggested antidotes, 2 antidotes reached consensus (sugammadex and prothrombin complex concentrate) while 5 did not reach consensus. These antidotes were reviewed in Round 3.

Round three of the e-Delphi study consisted of a reflection on the 2 antidotes that did reach consensus from Round 2, and a re-rating of the 5 antidotes that did not reach consensus (unithiol, glucarpidase, hyperbaric oxygen therapy, uridine triacetate and calcium trisodium). The expert panel confirmed their consensus on the inclusion of both sugammadex and prothrombin complex concentrate in the essential antidote list for Kuwait. However, they did not reach consensus on the inclusion of the 5 antidotes mentioned above in the essential list of antidotes in Kuwait–those 5 antidotes were therefore removed. Table 2 provides the consensus level for each antidote in each round.

**Table 2:** Consensus level of each antidote in each Delphi round.

No	Antidote	Toxic exposure	Round 1	Round 2	Round 3
			n = 22	n = 22	n = 20
1	Acetylcysteine IV	Acetaminophen	100%	-	-
2	Acetylcysteine PO	Acetaminophen	81.8%*	81.8%*	-
3	Activated charcoal PO	Oral poisons bound to charcoal	100%	-	-
4	Atropine sulfate	Organophosphorus bradycardia	100%	-	-
5	Calcium chloride	Calcium channel blockers, hypermagnesemia, hyperkalemia	100%	-	-
6	Calcium gluconate	Hydrofluoric acid burns	95.4%	-	-
7	Calcium gluconate gel	Hydrofluoric acid burns	95.4%	-	-
8	Dicobalt edetate	Cyanide	81.8%*	63.6%	-
9	Hydroxocobalamin (Cyanokit®)	Cyanide	100%	-	-
10	Sodium nitrite	Cyanide	81.8%	-	-
11	Sodium thiosulphate	Cyanide	81.8%	-	-
12	Flumazenil	Over-sedation with benzodiazepines	100%	-	-
13	Glucagon	Beta Blockers/Calcium Channel Blockers	100%	-	-
14	Dextrose	Calcium channel blockers cardiotoxicity reversal,	100%	-	-
15	Lipid Emulsion (Intralipid 20%)	Severe, systemic local anaesthetic toxicity	90.9%	-	-
16	Methylthioninium chloride (methylene blue)	Methaemoglobinaemia	100%	-	-
17	Naloxone (Narcan®)	Opioids	100%	-	-
18	Procyclidine injection	For Extrapyramidal symptoms	86.4%	-	-
19	Sodium bicarbonate	Tricyclic antidepressants	100%	-	-
20	Thiamine (Vit. B1)	Ethanol toxicity	90.9%	-	-
21	Antisnake antivenin	Snake venoms	95.4%	-	-
22	Antiscorpion antivenin	Scorpion venoms	95.4%	-	-
23	Black widow spider antivenin	Black widow spider venom	59.1%	68.2%	-
24	Bromocriptine mesylate (Parlodel®)	Drugs causing Neuroleptic Malignant Syndrome	86.4%	-	-
25	Calcium folinate	Methotrexate/Methanol toxicity	77.3%	-	-
26	Cyproheptadine	Drugs causing serotonin syndrome	86.4%	-	-
27	L-Carnitine	Valproic acid	91.0%	-	-
28	Dantrolene	Drugs causing Neuroleptic Malignant Syndrome	91.0%	-	-
29	Desferrioxamine (Desferal®)	Iron	100%	-	-
30	Digoxin specific antibody fragments fab	Digoxin	100%	-	-
31	Fomepizole	Ethylene glycol	100%	-	-
32	Ethanol IV	Ethylene glycol	72.7%	77.3%	-
33	Idarucizumab	Dabigatran	91.0%	-	-
34	PEG solution	Whole bowel irrigation	91.0%	-	-
35	Mesna	Cyclophosphamide	72.8%	63.6%	-
36	Octreotide acetate (Sandostatin)	Sulphonylureas, hypoglycemia	95.5%	-	-
37	Pralidoxime	Organophosphate insecticides	100%	-	-
38	Phentolamine	Digital ischaemia, Resistant hypertension	81.8%	-	-
39	Phytomenadione IV (Vitamin K1)	Warfarin	100%	-	-
40	Phytomenadione PO (Vitamin K1)	Warfarin	86.4%	-	-
41	Protamine sulphate	Heparin & low molecular weight heparins	100%	-	-
42	Pyridoxine (Vitamin B6)	Isoniazid seizures	95.4%	-	-
43	Calcium disodium EDTA	Heavy metals: particularly lead, zinc	81.8%	-	-
44	Physostigmine	Atropine	81.8%	-	-
45	Potassium iodide	Radioactive iodine	86.4%	-	-
46	Succimer (dimercaptosuccinic acid)	Lead and mercury	81.8%	-	-
47	Prussian blue	Thallium, radioactive cesium	72.8%	72.7%	-
48	Sugammadex	Neuromuscular blockade drugs	-	100%	-
49	Unithiol	Heavy metals, particularly mercury	-	72.7%	65%
50	Glucarpidase	Methotrexate	-	72.8%	65%
51	Hyperbaric Oxygen therapy	Carbon monoxide	-	86.4%*	85%*
52	Prothrombin complex concentrate	Reversal of acquired coagulation factor deficiency	-	95.4%	-
53	Uridine triacetate	fluorouracil or capecitabine overdose	-	59.1%	50%
54	Calcium trisodium	Contamination with plutonium, americium or curium	-	63.6%	50%

* Had a minimum of one expert with strong disagreement.

In total, 43 antidotes were included in the final list of essential antidotes for Kuwait. The final list of essential antidotes is provided in Table 3.

**Table 3:** Final list of essential antidotes for hospitals that provide emergency care services in Kuwait.

No.	Antidote	Toxic exposure
1	Acetylcysteine IV	Acetaminophen
2	Activated charcoal PO	Oral poisons bound to charcoal
3	Atropine sulfate	Organophosphorus bradycardia
4	Calcium chloride	Calcium channel blockers, hypermagnesemia hyperkalemia
5	Calcium gluconate	Hydrofluoric acid burns
6	Calcium gluconate gel	Hydrofluoric acid burns
7	Hydroxocobalamin (Cyanokit®)	Cyanide
8	Sodium nitrite	Cyanide
9	Sodium thiosulphate	Cyanide
10	Flumazenil	Over-sedation with benzodiazepines
11	Glucagon	For beta blockers/calcium channel blockers
12	Dextrose	Calcium Channel Blockers cardiotoxicity reversal
13	Lipid Emulsion (Intralipid 20%)	Severe, systemic local anaesthetic toxicity
14	Methylthioninium chloride (methylene blue)	Methaemoglobinaemia
15	Naloxone (Narcan®)	Opioids
16	Procyclidine injection	For extra-pyramidal symptoms
17	Sodium bicarbonate	Tricyclic antidepressants
18	Thiamine (Vit. B1)	Ethanol
19	Sugammadex	Neuromuscular blockade drug
20	Prothrombin complex concentrate	Reversal of acquired coagulation factor deficiency
21	Antisnake antivenin	Snake venoms
22	Antiscorpion antivenin	Scorpion venoms
23	Bromocriptine mesylate (Parlodel®)	Drugs causing Neuroleptic Malignant Syndrome
24	Calcium folinate	Methotrexate/Methanol
25	Cyproheptadine	Drugs causing serotonin syndrome
26	L-Carnitine	Valproic acid
27	Dantrolene	Drugs causing Neuroleptic Malignant Syndrome
28	Desferrioxamine (Desferal®)	Iron
29	Digoxin specific antibody fragments fab	Digoxin
30	Fomepizole	Ethylene glycol
31	Idarucizumab	Dabigatran
32	PEG solution (polyethylene glycol)	Whole bowel irrigation
33	Octreotide acetate (Sandostatin)	For sulphonylureas, hypoglycemia
34	Pralidoxime	Organophosphate insecticides
35	Phentolamine	Digital ischaemia, Resistant hypertension
36	Phytomenadione IV (Vitamin K1)	Warfarin
37	Phytomenadione PO (Vitamin K1)	Warfarin
38	Protamine sulphate	Heparin & low molecular weight heparins
39	Pyridoxine (Vit. B6)	For isoniazid seizures
40	Calcium disodium EDTA	Heavy metals particularly lead, zinc
41	Physostigmine	Atropine poisoning
42	Potassium iodide	Radioactive iodine
43	Succimer (dimercaptosuccinic acid)	Chelating agent for lead and mercury

## Discussion

Effective management of poisoning requires adequate stocking of antidotes in hospitals that provide emergency care. Antidote stocking represents a major challenge to hospitals all over the world, including Kuwait. It has been reported that public and private hospitals in Kuwait have suboptimal stocks of essential antidotes [8]. In order to assist hospitals to reduce costs and improve patient care, this study aimed to develop an essential antidote list for Kuwait through expert consensus using the e-Delphi method. To our knowledge, this study is the first study to use the e-Delphi technique in the Europe, Middle East and Africa (EMEA) region to generate a nationally relevant list of antidotes by multidisciplinary expert consensus, to secure adequate availability of antidotes in emergency care hospitals.

One of the characteristics of the Delphi method for reaching consensus is that it does not generate right or wrong answers or any definitive answers but rather, a valid expert opinion on each standard [28]. It has the advantage of including large, diverse groups, that are geographically dispersed, to obtain a reliable data through a large number of viewpoints, while preserving anonymity [10]. One unique advantage of the Delphi technique is the provision of controlled feedback to the expert panel, where obtained data is analyzed and presented in an easily interpretable format to all the experts after every round. This controlled feedback ensures that the experts view their previous results against the group results and have the opportunity to change their responses if needed. This ensures stability of results when final closure is done [29]. Specifically, the e-Delphi method is beneficial whenever the judgment of experts is needed but face-to-face interaction is not feasible.

Our expert panel consisted of 22 experts in Round 1 and 2, and 20 experts in Round 3 (9% dropout rate). Acceptable dropout rates in Delphi studies have been reported to be 20% across three rounds of consensus development [21, 30, 31]. In addition, literature on Delphi studies shows that a minimum of twelve experts is generally considered acceptable to enable consensus achievement, as Delphi sample sizes depend more on group diversity in reaching consensus than statistical power [32]. This highlights the reason why our purposive sample of 22 experts was needed, as they were geographically dispersed around the healthcare regions in Kuwait, and from both the public and private sector with diverse specialties.

Our study’s preliminary antidote list (that contained 47 antidotes) was based on the results of a national audit of antidotes in Kuwait that looked at the availability of antidotes in public and private hospitals that provide emergency care in 2020 [8]. In this study, Round 1 resulted in 41 antidotes out of 47 antidotes to be included in the final list of essential antidotes for Kuwait, while Round 2 and 3 provided consensus on two of the newly suggested antidotes by the expert panel, giving a final list of 43 antidotes. A total of 11 antidotes did not reach consensus in this study, despite going through at least 2 rounds. Reasons behind some antidotes not reaching consensus are: i) less costly alternatives available (e.g IV acetylcysteine more affordable than PO) [33], ii) a more acceptable (more tolerable) alternative available (e.g. IV acetylcysteine more tolerable than PO, with shorter course of therapy) [33], iii) risky side effects (e.g. dicobalt edetate and black widow spider antivenin) [34–37] iv) sub-specialized need (e.g. mesna needed for cyclophosphamide toxicity, which is used mostly in cancer care) [34] and v) world-wide unavailability (e.g. Prussian blue) [38].

Two new antidotes reached the consensus to be added to the essential antidotes list in Kuwait, these were: sugammadex and prothrombin complex concentrate. Sugammadex is a novel antidote used for the immediate reversal of neuromuscular blockade induced by aminosteroid muscle relaxants such as rocuronium, vecuronium and pancuronium [39]. The expert panel also agreed on the inclusion of prothrombin complex concentrate (PCC) in the essential antidote list for Kuwait. PCC, indicated for rapid anticoagulation reversal of vitamin K antagonists particularly in the emergent setting (e.g., intracranial hemorrhage or a need for urgent invasive surgery), has been recommended over the classical treatment with fresh frozen plasma (FFP) and vitamin K owing to the smaller volume of PCC needed compared to FFP [40]. Another indication for PCC, although not FDA approved yet, is the reversal of direct oral anticoagulants (DOAC)-induced anticoagulation when a more specific antidote is unavailable [41].

The list of essential antidotes is a tool that would support healthcare facilities to be better prepared in the management of poisoned patients in emergency care departments as the availability of an antidotes essential list will help emergency physicians and other healthcare providers to provide optimum and timely care for patients with risk of poisoning. Emergency care and hospital managers may aim to ensure appropriate stocks are available of these essential antidotes. Future work has to be conducted to generate a minimum stock level to treat an adult patient for 24 hours, as well as cost-effectiveness studies. Pilot testing of the final list of essential antidotes before widespread implementation would ensure optimal feasibility and applicability. In addition, this essential list of antidotes would need to be updated regularly to take into consideration any changes in antidote needs by the poisoned population and availability or supply.

This is the first study in Kuwait that aims to provide a useful and clinically relevant list of essential antidotes based on an expert consensus for hospitals that provide emergency in Kuwait. The main strength of this study is the use of the e-Delphi method, as it proved to be ideal in establishing expert consensus on our study subject, and is a valid method to establish consensus for development of a national essential antidote list and can be used in different countries. Development of a national essential antidote list required a process that is evidence-based as well as consultative. We believe our methodology has displayed a model that integrated best evidence [1, 20] with a consultative process for consensus development–the e-Delphi method. The e-Delphi survey, conducted over three rounds with experts from diverse care settings, ensured a reliable collection of professional inputs on this important subject matter. One advantage of the Delphi method is that it offers anonymity which helps to eliminate the fear or influence of a dominating expert [42] which can lead to erroneous data collection and defective consensus [43]. In particular, the use of the e-Delphi method has been proven to remove certain biases that are observed with other face-to-face consensus processes such as the nominal group approach [44]. Performing a local/regional Delphi study will ensure that all countries have the required stock and type of antidote to meet their local needs. Ideally, hospitals should conduct their own HVA to determine their antidote requirements, however, as mentioned earlier, this can be time intensive and costly.

One of the limitations of this study is that there are not enough updated epidemiological studies for the incidence of poisoning in Kuwait. This can justify the reason for some antidotes not reaching consensus or having at least a strongly disagree answer such as mesna, unithiol and CA-DTPA. The need to conduct a hazard vulnerability assessment in hospitals of Kuwait is paramount to understand the scale of poisoning locally.

## Conclusions

This study has highlighted the importance of development of an essential antidote list for the management of poisoned patients by physicians at emergency care departments in Kuwait through a reliable, iterative process using the e-Delphi method. It reemphasizes the need for continuous planning and evaluation of the local needs of antidotes for poisoning cases in countries, by the successful collaboration of academic expertise with clinical practitioners’ experience to develop the most appropriate evidence-based framework. The optimal approach for ensuring appropriate stocking of antidotes and, hence, successful management of poisoning would be the utilization of the recommendations from the expert panel consensus together with an antidote hazard vulnerability assessment specific for each hospital. Future work should determine the applicability of the list across hospitals in Kuwait and the impact of list adoption on the management of actual poisoning cases.

## Supporting information

S1 File (PDF)

S2 File (PDF)

S3 File (PDF)
