# Supplementary material for: Expert consensus for a national essential antidote list: E-Delphi method
Source: PLoS One. 2022 Jun 16;17(6):e0269456. doi: 10.1371/journal.pone.0269456 (PMC9202922; doi:10.1371/journal.pone.0269456)
Supplement: S1 File — (PDF) [file pone.0269456.s001.pdf]

Default Report

Antidotes stocking in the Hospitals (Agreement only)

April 28th 2020, 5:28 pm EAT

- - Please note that you have the right to accept or to reject to participate in this study. In case you accepted, you are free to withdraw at any time without affecting your professional performance. This survey will take you approximately 20-30 minutes to complete. If you would like to have any further information, please do not hesitate to contact me. Yours sincerely, Sara Al-Ansari Final year pharmacy student Email: sara.alansari@hsc.edu.kw Telephone: 65633350 Dalal Al-Taweel Director, Kuwait Medicines Information Centre (KUMIC), Faculty of Pharmacy, Kuwait University. Email: d.altaweel@hsc.edu.kw Telephone: (+965) 24636897

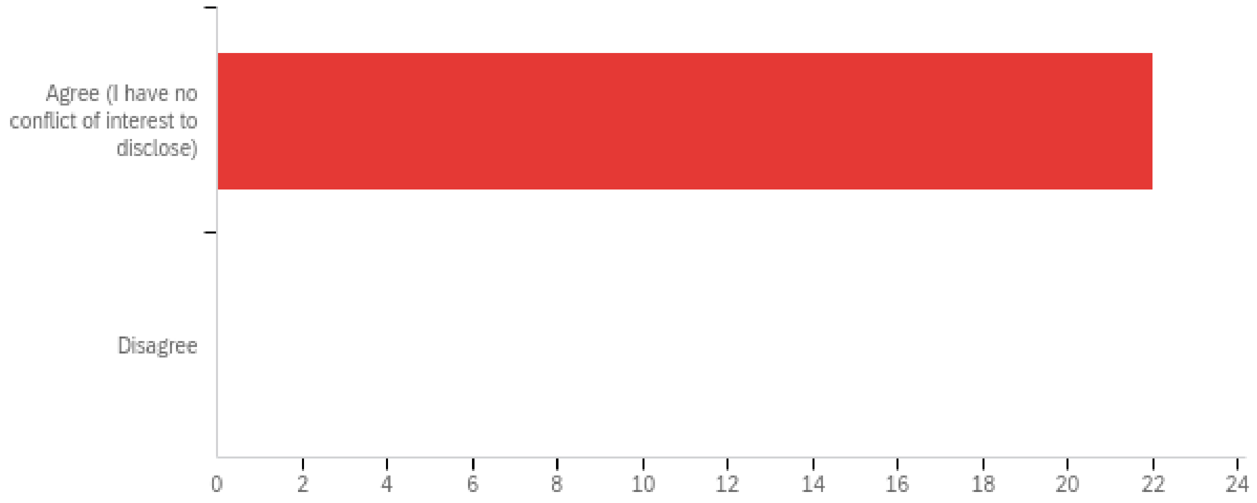

#	Field	Minimum	Maximum	Mean	Std Deviation	Variance	Count
1	Please note that you have the right to accept or to reject to participate in this study. In case you accepted, you are free to withdraw at any time without affecting your professional performance. This survey will take you approximately 20-30 minutes to complete. If you would like to have any further information, please do not hesitate to contact me. Yours sincerely, Sara Al-Ansari Final year pharmacy student Email: sara.alansari@hsc.edu.kw Telephone: 65633350 Dalal Al-Taweel Director, Kuwait Medicines	1.00	1.00	1.00	0.00	0.00	22

Information Centre (KUMIC), Faculty of Pharmacy, Kuwait University. Email: d.altaweel@hsc.edu.kw Telephone: (+965) 24636897					

#	Answer	%	Count
1	Agree (I have no conflict of interest to disclose)	100.00%	22
2	Disagree	0.00%	0
	Total	100%	22

Q1 - 1) Participant full name:

1) Participant full name:
Nadeem Abdulaziz Alduaij
Laila shehab ahmed
Mariam Mahmoud Ibrahim
Mohammad hajeyah
Fatoumah Alabdulrazzaq
Mohama hatem
Ali mohsen
Huda Ahmad Sadeq
MOHAMMAD ALZAYED
Ahmad Wajih Alqallaf
Faisal Alghanem
Abdulrahman ali Husain
essam nabeel alayoub
Adnan Hajjiah
Abdullah
Haider Ahmad
Selma Alqattan
Hussein Faidhi Aldulaimi
Amira
Abdulaziz Alburaidi
Ahmad alshadad
Dr Abdullatif Aloumi

Q2 - 2) Site of practice: (tick all that apply)

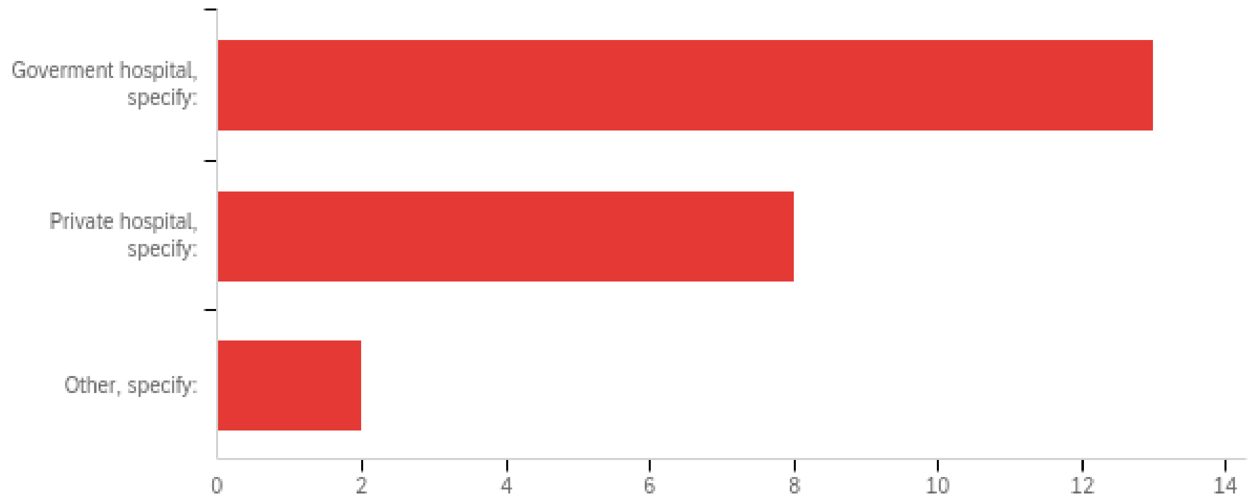

#	Answer	%	Count
1	Goverment hospital, specify:	56.52%	13
2	Private hospital, specify:	34.78%	8
3	Other, specify:	8.70%	2
	Total	100%	23

Q2_1_TEXT - Goverment hospital, specify:

Goverment hospital, specify: - Text
Amiri Hospital
Jaber Hospital
Pediatric Emergency
Jaber Al Ahmad Hospital
Farwaniya hospital
Al-Sabah Hospital
Jahra Hospital
Amiri Hospital
Jaber alahmed hospital

Farwaniya Hospital
MKH
Amiri

Q2_2_TEXT - Private hospital, specify:

Private hospital, specify: - Text
Ahmadi Hospital
Taiba
Ahmadi Hospital
taibahhospital
Ahmadi Hospital
Taiba Hospital
Taiba Hospital

Q2_3_TEXT - Other, specify:

Other, specify: - Text
private enterprise

Q3 - 3) Specialty: (tick all that apply)

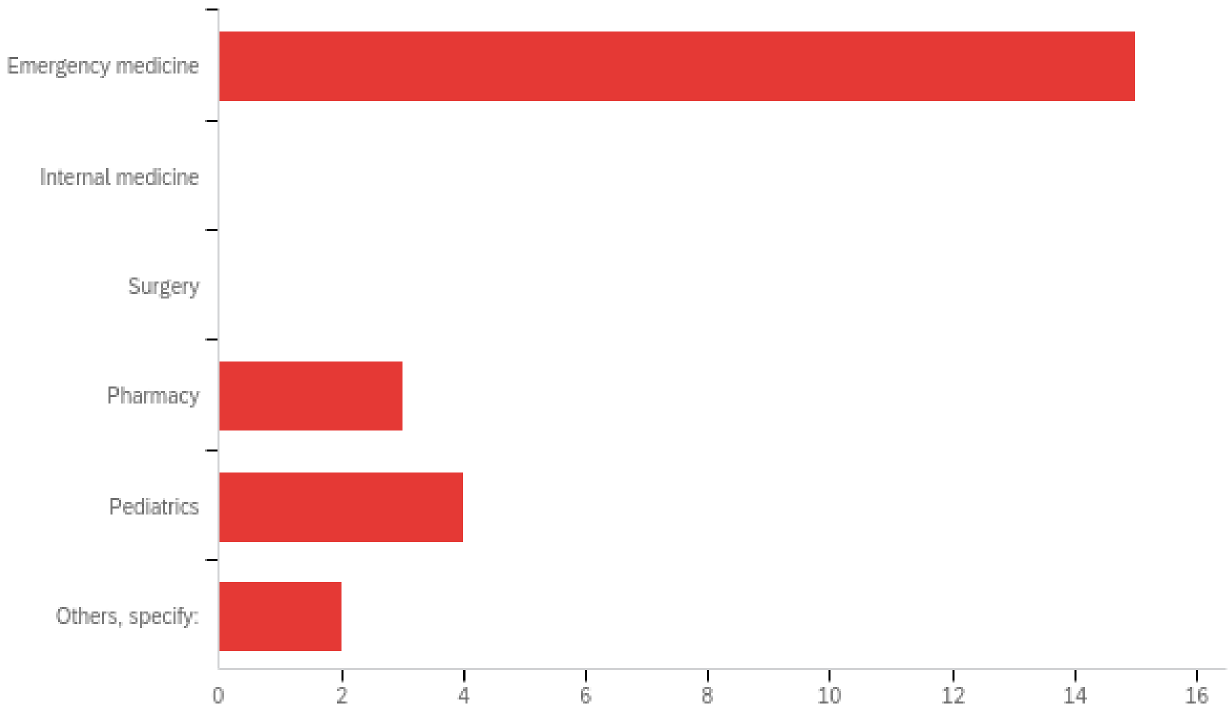

#	Answer	%	Count
1	Emergency medicine	62.50%	15
2	Internal medicine	0.00%	0
3	Surgery	0.00%	0
4	Pharmacy	12.50%	3
5	Pediatrics	16.67%	4
6	Others, specify:	8.33%	2
	Total	100%	24

Q3_6_TEXT - Others, specify:

Others, specify: - Text
Medical toxicology
Anesthesia and Intensive care

Q4 - 4) Position:

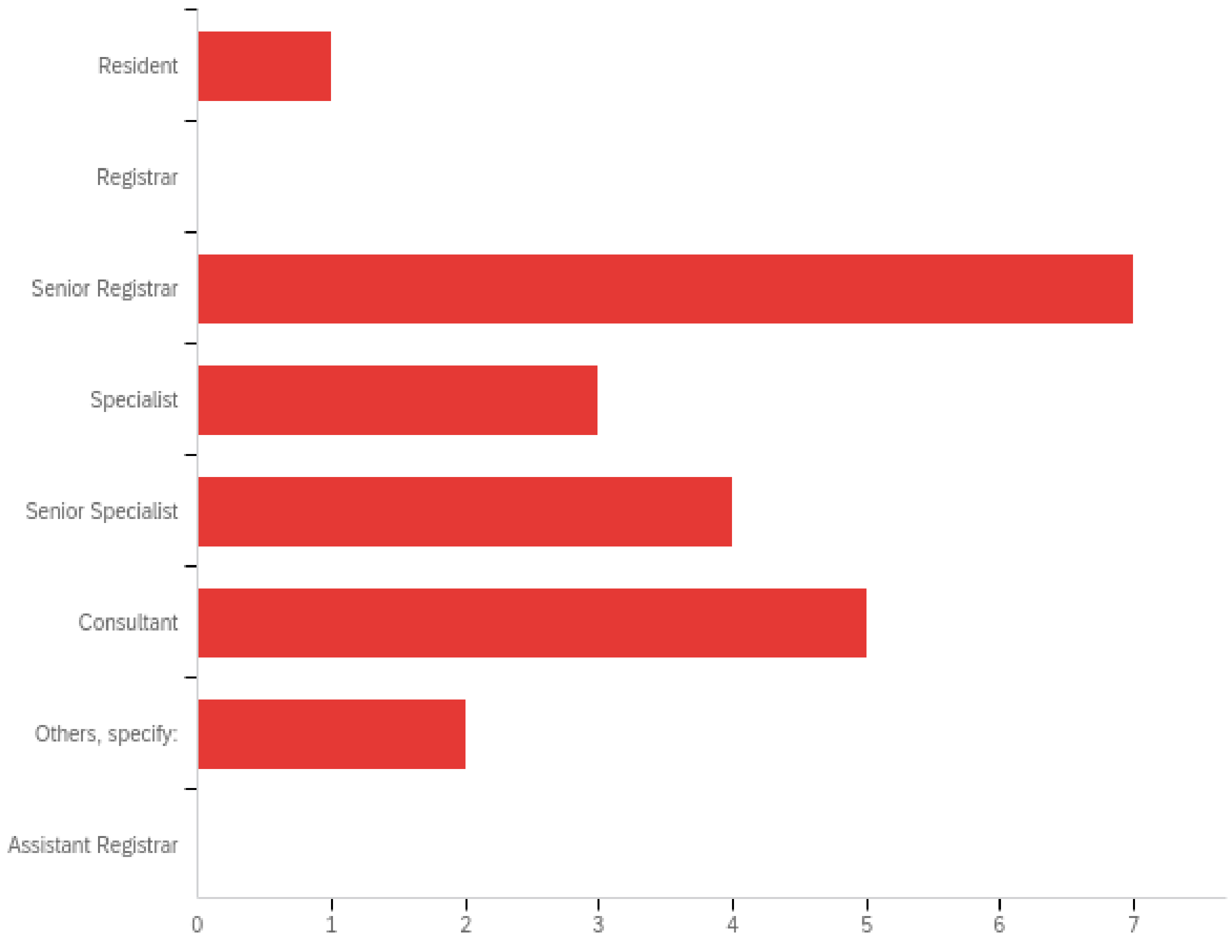

#	Field	Minimum	Maximum	Mean	Std Deviation	Variance	Count
1	4) Position: - Selected Choice	1.00	7.00	4.45	1.56	2.43	22

#	Answer	%	Count
1	Resident	4.55%	1
2	Registrar	0.00%	0
3	Senior Registrar	31.82%	7
4	Specialist	13.64%	3
5	Senior Specialist	18.18%	4

6	Consultant	22.73%	5
7	Others, specify:	9.09%	2
8	Assistant Registrar	0.00%	0
	Total	100%	22

Q4_7_TEXT - Others, specify:

Others, specify: - Text
Clinical Pharmacist
Pharmacist

Q5 - 5) In your opinion, does Kuwait require its own expert-consensus antidote guidelines for adequate availability of antidotes for proper management of poisoning in Kuwait?

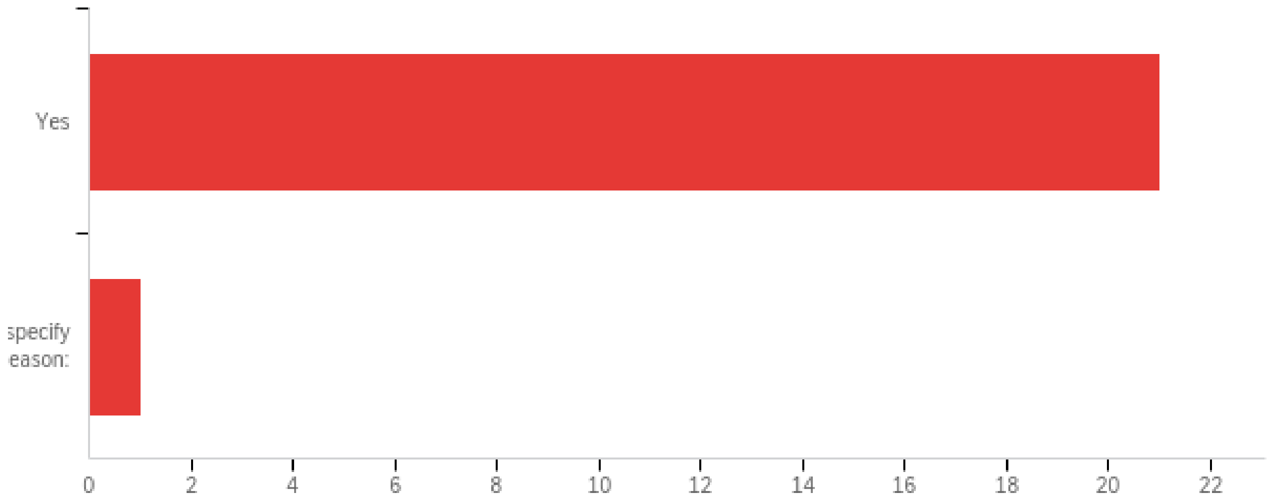

#	Field	Minimum	Maximum	Mean	Std Deviation	Variance	Count
1	5) In your opinion, does Kuwait require its own expert-consensus antidote guidelines for adequate availability of antidotes for proper management of poisoning in Kuwait? - Selected Choice	1.00	2.00	1.05	0.21	0.04	22

#	Answer	%	Count
1	Yes	95.45%	21
2	No, specify reason:	4.55%	1
	Total	100%	22

Q5_2_TEXT - No, specify reason:

No, specify reason: - Text
Given the landscape, a regional guideline may be more adequate

Q6 - 6) To your knowledge, does a national Kuwaiti antidote availability guideline exist?

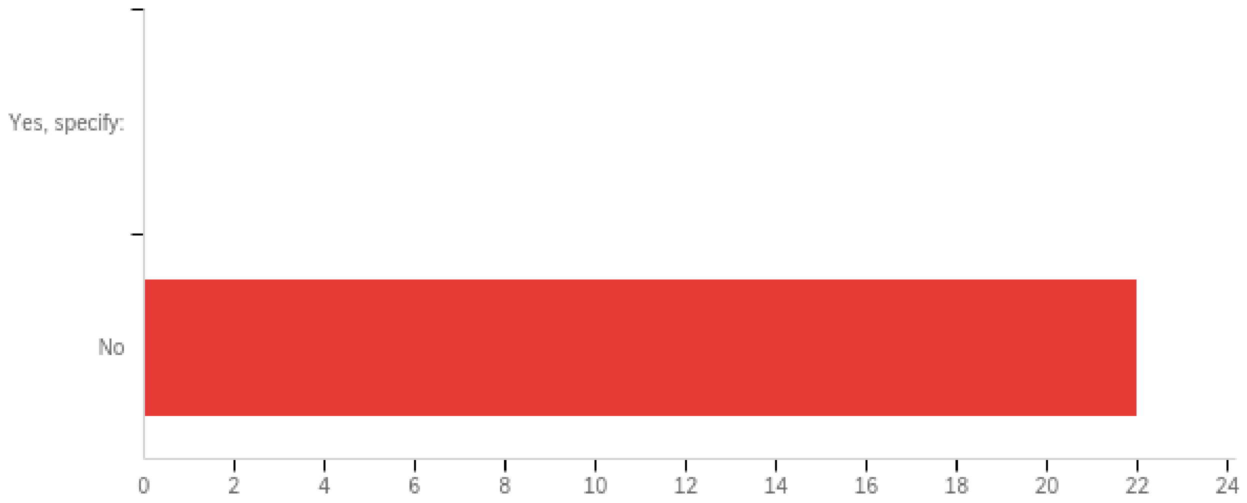

#	Field	Minimum	Maximum	Mean	Std Deviation	Variance	Count
1	6) To your knowledge, does a national Kuwaiti antidote availability guideline exist? - Selected Choice	2.00	2.00	2.00	0.00	0.00	22

#	Answer	%	Count
1	Yes, specify:	0.00%	0
2	No	100.00%	22
	Total	100%	22

Q6_1_TEXT - Yes, specify:

Yes, specify: - Text

Q7 - 7) What antidote guideline do you currently follow?

7) What antidote guideline do you currently follow?
Not currently practicing
Sick kids hospital
from uptodate, AAP guidelines
Royal college of emergency medicine
Text books and online studies
Don’t know
Emergency medicine references
American Academy of Clinical Toxicology practice guidelines
Reference book of poisonings
I am using the BNF providing that the agent suggested is available...
British
ACEP, Other international tox society
ACEP
Marryland Poison Centre
.
International guidelines
Lexicomp and Micromedex
ACMT/NYPCC recommendations
RCEMNPISA
ACEP Toxicology Antidote Guideline (App) and RCEM Guidelines
Local department
Expert Consensus Guidelines for Stocking of Antidotes in Hospitals That Provide Emergency Care

Q18 - 8) For the inclusion of each antidote in the Kuwait National Antidote GuidelinesPlease select whether you (strongly agree / agree / disagree / strongly disagree)Note: If you have any comment about any antidote you can comment in the box beside it.

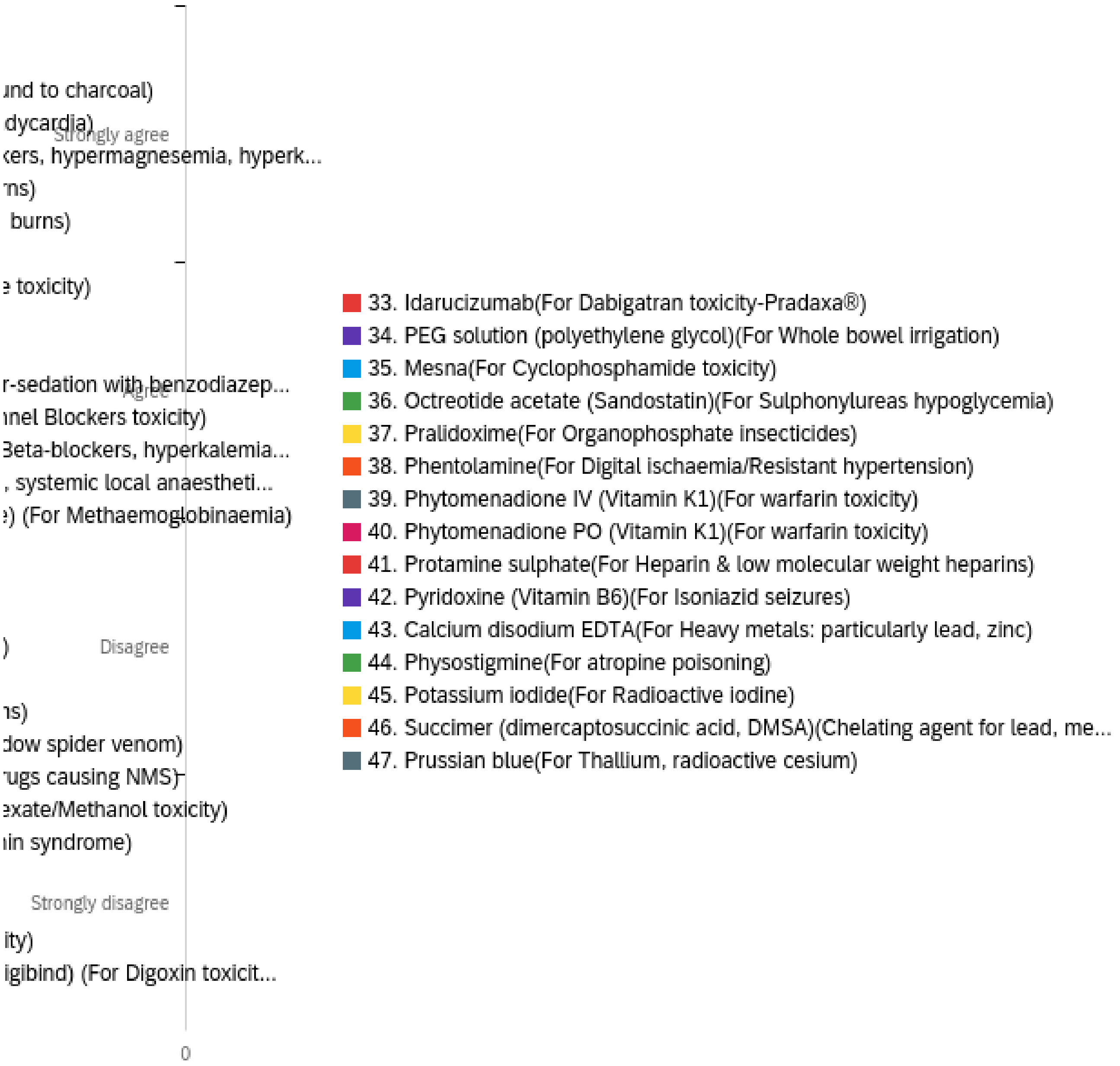

#	Field	Minimum	Maximum	Mean	Std Deviation	Variance	Count
1	1. Acetylcysteine IV (For Acetaminophen)	26.00	27.00	26.09	0.29	0.08	22
2	2. Acetylcysteine PO (For Acetaminophen)	26.00	30.00	26.91	1.20	1.45	22
3	3. Activated charcoal oral (For oral poisons bound to charcoal)	26.00	27.00	26.23	0.42	0.18	22

4	4. Atropine sulfate (For Organophosphorus/Bradycardia)	26.00	27.00	26.05	0.21	0.04	22
5	5. Calcium chloride (For Calcium channel blockers, hypermagnesemia, hyperkalemia)	26.00	27.00	26.18	0.39	0.15	22
6	6. Calcium gluconate (For hydrofluoric acid burns)	26.00	29.00	26.36	0.71	0.50	22
7	7. Calcium gluconate gel (For hydrofluoric acid burns)	26.00	29.00	26.55	0.72	0.52	22
8	8. Dicobalt edetate (For Cyanide toxicity)	26.00	30.00	27.09	1.24	1.54	22
9	9. Hydroxocobalamin (Cyanokit®) (For Cyanide toxicity)	26.00	27.00	26.18	0.39	0.15	22
10	10. Sodium nitrite (For Cyanide toxicity)	26.00	29.00	26.91	1.08	1.17	22
11	11. Sodium thiosulphate (For Cyanide toxicity)	26.00	29.00	26.86	1.10	1.21	22
12	12. Flumazenil (For Reversal of iatrogenic over-sedation with benzodiazepines)	26.00	27.00	26.36	0.48	0.23	22
13	13. Glucagon (For Beta Blockers/Calcium Channel Blockers toxicity)	26.00	27.00	26.18	0.39	0.15	22
14	14. Dextrose (For CCB cardiotoxicity reversal, Beta-blockers, hyperkalemia)	26.00	27.00	26.14	0.34	0.12	22
15	15. Lipid Emulsion (Intralipid 20%) (For Severe, systemic local anaesthetic toxicity)	26.00	29.00	26.64	0.88	0.78	22
16	16. Methylthioninium chloride (methylene blue) (For Methaemoglobinaemia)	26.00	27.00	26.27	0.45	0.20	22
17	17. Naloxone (Narcan®) (For Opioids toxicity)	26.00	27.00	26.09	0.29	0.08	22
18	18. Procyclidine injection (For EPS symptoms)	26.00	29.00	26.82	0.98	0.97	22
19	19. Sodium bicarbonate (For TCAs)	26.00	27.00	26.09	0.29	0.08	22
20	20. Thiamine (Vitamin B1) (For Ethanol toxicity)	26.00	29.00	26.50	0.89	0.80	22
21	21. Antisnake antivenin (For Snake venoms)	26.00	29.00	26.41	0.72	0.51	22
22	22. Antiscorpion antivenin (For Scorpion venoms)	26.00	29.00	26.50	0.72	0.52	22
23	23. Black widow spider antivenin (For Black widow spider venom)	26.00	30.00	27.55	1.37	1.88	22
24	24. Bromocriptine mesylate (Parlodel®) (For drugs causing NMS)	26.00	29.00	26.91	0.95	0.90	22
25	25. Calcium folinate (Leucovorin) (For Methotrexate/Methanol toxicity)	26.00	29.00	27.05	1.15	1.32	22
26	26. Cyproheptadine (For drugs causing serotonin syndrome)	26.00	29.00	26.86	0.97	0.94	22
27	27. L-Carnitine (For Valproic acid toxicity)	26.00	29.00	26.73	0.86	0.74	22
28	28. Dantrolene (For drugs causing NMS)	26.00	29.00	26.73	0.86	0.74	22

29	29. Desferrioxamine (Desferal®) (For Iron toxicity)	26.00	27.00	26.14	0.34	0.12	22
30	30. Digoxin specific antibody fragments fab (Digibind) (For Digoxin toxicity)	26.00	27.00	26.14	0.34	0.12	22
31	31. Fomepizole (For Ethylene glycol toxicity)	26.00	27.00	26.14	0.34	0.12	22
32	32. Ethanol IV (For Ethylene glycol toxicity)	26.00	30.00	27.14	1.32	1.75	22
33	33. Idarucizumab (For Dabigatran toxicity-Pradaxa®)	26.00	29.00	26.68	0.87	0.76	22
34	34. PEG solution (polyethylene glycol) (For Whole bowel irrigation)	26.00	29.00	26.64	0.88	0.78	22
35	35. Mesna (For Cyclophosphamide toxicity)	26.00	29.00	27.23	1.17	1.36	22
36	36. Octreotide acetate (Sandostatin) (For Sulphonylureas hypoglycemia)	26.00	29.00	26.50	0.72	0.52	22
37	37. Pralidoxime (For Organophosphate insecticides)	26.00	27.00	26.32	0.47	0.22	22
38	38. Phentolamine (For Digital ischaemia/Resistant hypertension)	26.00	29.00	26.86	1.10	1.21	22
39	39. Phytomenadione IV (Vitamin K1) (For warfarin toxicity)	26.00	27.00	26.09	0.29	0.08	22
40	40. Phytomenadione PO (Vitamin K1) (For warfarin toxicity)	26.00	29.00	26.64	1.02	1.05	22
41	41. Protamine sulphate (For Heparin & low molecular weight heparins)	26.00	27.00	26.32	0.47	0.22	22
42	42. Pyridoxine (Vitamin B6) (For Isoniazid seizures)	26.00	29.00	26.45	0.72	0.52	22
43	43. Calcium disodium EDTA (For Heavy metals: particularly lead, zinc)	26.00	29.00	26.86	1.10	1.21	22
44	44. Physostigmine (For atropine poisoning)	26.00	29.00	26.73	1.14	1.29	22
45	45. Potassium iodide (For Radioactive iodine)	26.00	29.00	26.77	1.00	0.99	22
46	46. Succimer (dimercaptosuccinic acid, DMSA) (Chelating agent for lead, mercury)	26.00	29.00	26.91	1.08	1.17	22
47	47. Prussian blue (For Thallium, radioactive cesium)	26.00	29.00	27.18	1.19	1.42	22

#	Question	Strongly agree		Agree		Disagree		Strongly disagree		Total
1	1. Acetylcysteine IV (For Acetaminophen)	90.91%	20	9.09%	2	0.00%	0	0.00%	0	22
2	2. Acetylcysteine PO (For Acetaminophen)	50.00%	11	31.82%	7	13.64%	3	4.55%	1	22
3	3. Activated charcoal oral (For oral poisons bound to charcoal)	77.27%	17	22.73%	5	0.00%	0	0.00%	0	22
4	4. Atropine sulfate (For Organophosphorus/Bradycardia)	95.45%	21	4.55%	1	0.00%	0	0.00%	0	22
5	5. Calcium chloride (For Calcium channel blockers, hypermagnesemia, hyperkalemia)	81.82%	18	18.18%	4	0.00%	0	0.00%	0	22
6	6. Calcium gluconate (For hydrofluoric acid burns)	72.73%	16	22.73%	5	4.55%	1	0.00%	0	22
7	7. Calcium gluconate gel (For hydrofluoric acid burns)	54.55%	12	40.91%	9	4.55%	1	0.00%	0	22
8	8. Dicobalt edetate (For Cyanide toxicity)	36.36%	8	45.45%	10	9.09%	2	9.09%	2	22
9	9. Hydroxocobalamin (Cyanokit®) (For Cyanide toxicity)	81.82%	18	18.18%	4	0.00%	0	0.00%	0	22
10	10. Sodium nitrite (For Cyanide toxicity)	45.45%	10	36.36%	8	18.18%	4	0.00%	0	22
11	11. Sodium thiosulphate (For Cyanide toxicity)	50.00%	11	31.82%	7	18.18%	4	0.00%	0	22
12	12. Flumazenil (For Reversal of iatrogenic over-sedation with benzodiazepines)	63.64%	14	36.36%	8	0.00%	0	0.00%	0	22
13	13. Glucagon (For Beta Blockers/Calcium Channel Blockers toxicity)	81.82%	18	18.18%	4	0.00%	0	0.00%	0	22
14	14. Dextrose (For CCB cardiotoxicity reversal, Beta-blockers, hyperkalemia)	86.36%	19	13.64%	3	0.00%	0	0.00%	0	22
15	15. Lipid Emulsion (Intralipid 20%) (For Severe, systemic local anaesthetic toxicity)	54.55%	12	36.36%	8	9.09%	2	0.00%	0	22
16	16. Methylthioninium chloride (methylene blue) (For Methaemoglobinaemia)	72.73%	16	27.27%	6	0.00%	0	0.00%	0	22
17	17. Naloxone (Narcan®) (For Opioids toxicity)	90.91%	20	9.09%	2	0.00%	0	0.00%	0	22
18	18. Procyclidine injection (For EPS symptoms)	45.45%	10	40.91%	9	13.64%	3	0.00%	0	22
19	19. Sodium bicarbonate (For TCAs)	90.91%	20	9.09%	2	0.00%	0	0.00%	0	22
20	20. Thiamine (Vitamin B1) (For Ethanol toxicity)	68.18%	15	22.73%	5	9.09%	2	0.00%	0	22
21	21. Antisnake antivenin (For Snake venoms)	68.18%	15	27.27%	6	4.55%	1	0.00%	0	22

22	22. Antiscorpion antivenin (For Scorpion venoms)	59.09%	13	36.36%	8	4.55%	1	0.00%	0	22
23	23. Black widow spider antivenin (For Black widow spider venom)	31.82%	7	27.27%	6	36.36%	8	4.55%	1	22
24	24. Bromocriptine mesylate (Parlodel®) (For drugs causing NMS)	36.36%	8	50.00%	11	13.64%	3	0.00%	0	22
25	25. Calcium folinate (Leucovorin) (For Methotrexate/Methanol toxicity)	40.91%	9	36.36%	8	22.73%	5	0.00%	0	22
26	26. Cyproheptadine (For drugs causing serotonin syndrome)	40.91%	9	45.45%	10	13.64%	3	0.00%	0	22
27	27. L-Carnitine (For Valproic acid toxicity)	45.45%	10	45.45%	10	9.09%	2	0.00%	0	22
28	28. Dantrolene (For drugs causing NMS)	45.45%	10	45.45%	10	9.09%	2	0.00%	0	22
29	29. Desferrioxamine (Desferal®) (For Iron toxicity)	86.36%	19	13.64%	3	0.00%	0	0.00%	0	22
30	30. Digoxin specific antibody fragments fab (Digibind) (For Digoxin toxicity)	86.36%	19	13.64%	3	0.00%	0	0.00%	0	22
31	31. Fomepizole (For Ethylene glycol toxicity)	86.36%	19	13.64%	3	0.00%	0	0.00%	0	22
32	32. Ethanol IV (For Ethylene glycol toxicity)	45.45%	10	27.27%	6	22.73%	5	4.55%	1	22
33	33. Idarucizumab (For Dabigatran toxicity-Pradaxa®)	50.00%	11	40.91%	9	9.09%	2	0.00%	0	22
34	34. PEG solution (polyethylene glycol) (For Whole bowel irrigation)	54.55%	12	36.36%	8	9.09%	2	0.00%	0	22
35	35. Mesna (For Cyclophosphamide toxicity)	31.82%	7	40.91%	9	27.27%	6	0.00%	0	22
36	36. Octreotide acetate (Sandostatin) (For Sulphonylureas hypoglycemia)	59.09%	13	36.36%	8	4.55%	1	0.00%	0	22
37	37. Pralidoxime (For Organophosphate insecticides)	68.18%	15	31.82%	7	0.00%	0	0.00%	0	22
38	38. Phentolamine (For Digital ischaemia/Resistant hypertension)	50.00%	11	31.82%	7	18.18%	4	0.00%	0	22
39	39. Phytomenadione IV (Vitamin K1) (For warfarin toxicity)	90.91%	20	9.09%	2	0.00%	0	0.00%	0	22
40	40. Phytomenadione PO (Vitamin K1) (For warfarin toxicity)	63.64%	14	22.73%	5	13.64%	3	0.00%	0	22
41	41. Protamine sulphate (For Heparin & low molecular weight heparins)	68.18%	15	31.82%	7	0.00%	0	0.00%	0	22
42	42. Pyridoxine (Vitamin B6) (For Isoniazid seizures)	63.64%	14	31.82%	7	4.55%	1	0.00%	0	22
43	43. Calcium disodium EDTA (For Heavy metals: particularly lead, zinc)	50.00%	11	31.82%	7	18.18%	4	0.00%	0	22
44	44. Physostigmine (For atropine poisoning)	63.64%	14	18.18%	4	18.18%	4	0.00%	0	22
45	45. Potassium iodide (For Radioactive iodine)	50.00%	11	36.36%	8	13.64%	3	0.00%	0	22

46	46. Succimer (dimercaptosuccinic acid, DMSA) (Chelating agent for lead, mercury)	45.45%	10	36.36%	8	18.18%	4	0.00%	0	22
47	47. Prussian blue (For Thallium, radioactive cesium)	36.36%	8	36.36%	8	27.27%	6	0.00%	0	22

Q8 - Comments:

Unable to export widget. Please contact Qualtrics Support.

Q9 - 9) Do you recommend any other antidote(s) to be added to this list?

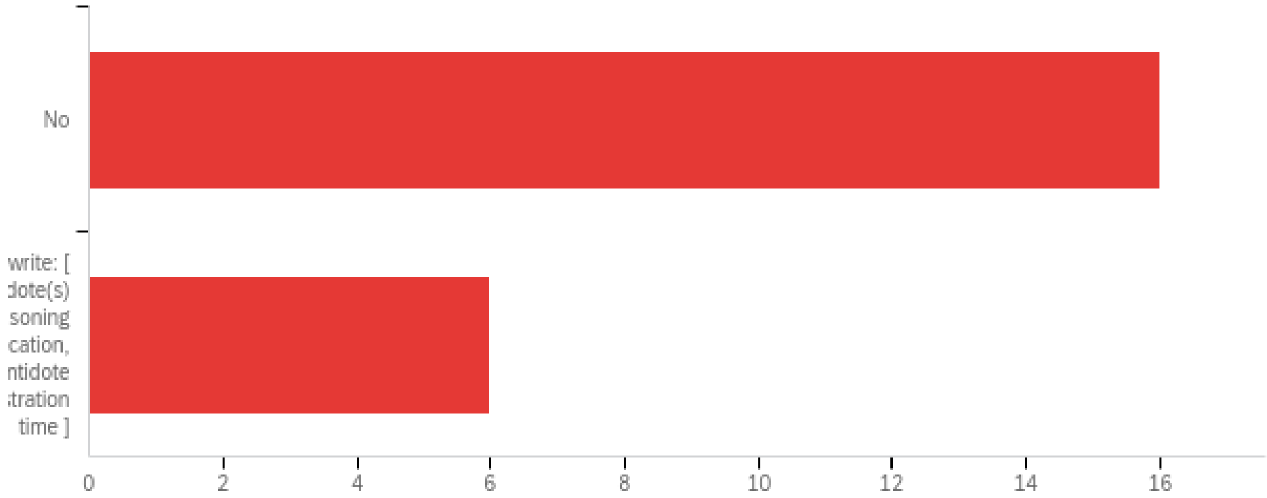

#	Field	Minimum	Maximum	Mean	Std Deviation	Variance	Count
1	9) Do you recommend any other antidote(s) to be added to this list? - Selected Choice	1.00	2.00	1.27	0.45	0.20	22

#	Answer	%	Count
1	No	72.73%	16
2	Yes, write: [Antidote(s) name, Poisoning indication, Antidote administration time]	27.27%	6
	Total	100%	22

Q9_2_TEXT - Yes, write: [Antidote(s) name, Poisoning indication, Antidote administrati...

Yes, write: [Antidote(s) name, Poisoning indication, Antidote administration time] - Text
[4-factor prothrombin complex concentrate, reversal of vitamin K antagonists], [Glucarpidase, methotrexate toxicity]
Unithiol(DMPS)for heavy metals(particularly mercury)
Sugammadex for neuromuscular blockade drugs
Sugamedex
Hyperbaric oxygen therapy for carbon monoxide toxicity
1: Calcium trisodium- (Internal contamination with plutonium, americium, or curium) pentetate (calcium DTPA)+ 2: prothrombin complex concentrate (Reversal of acquired coagulation factor deficiency induced by vitamin K antagonists) 3: Uridine triacetate (Fluorouracil or capecitabine overdose)
