# Supplementary material for: Expert consensus for a national essential antidote list: E-Delphi method
Source: PLoS One. 2022 Jun 16;17(6):e0269456. doi: 10.1371/journal.pone.0269456 (PMC9202922; doi:10.1371/journal.pone.0269456)
Supplement: S2 File — (PDF) [file pone.0269456.s002.pdf]

Default Report

Round 2 (Agreement only)

July 1, 2020 1:47 PM EAT

Intro - Dear Expert Panel Member, Thank you for returning the first round Delphi questionnaire. Your input makes us one step closer to the development of antidote stocking guidelines for Kuwait. You will now find the second (and final) round Delphi questionnaire which includes the responses of the whole expert panel towards the inclusion of each antidote into the Kuwaiti guidelines. In summary: Out of 47 antidotes, 41 were found to reach consensus (>75% agreement by all experts) 6 antidotes did not reach consensus (achieved 7 new antidotes were suggested to be added to the list. Your task now is: To reconsider your responses to all the antidotes that have reached consensus (and to re-assess them if you would like to change your response). To re-assess the six antidotes that did not reach consensus. To rate your agreement on the inclusion of 7 new antidotes to the national antidote guidelines. Please complete the Delphi questionnaire as fully as you can. Thank you for your continued participation in this study. Yours sincerely,

Sara Al-Ansari Final year pharmacy student Email: sara.alansari@hsc.edu.kw Telephone: (+965) 65633350 Dalal Al-Taweel Director, Kuwait Medicines Information Centre (KUMIC), Faculty of Pharmacy, Kuwait University. Email: d.altaweel@hsc.edu.kw Telephone: (+965) 24636897

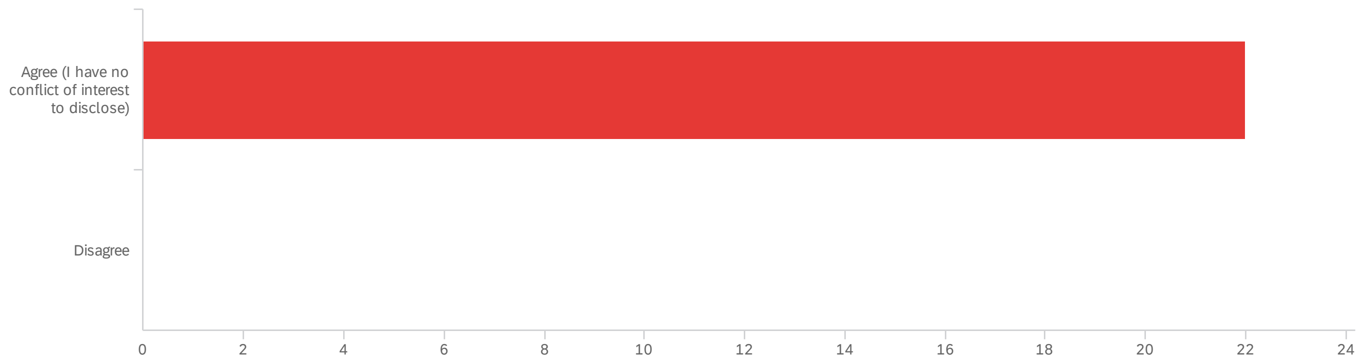

#	Field	Minimum	Maximum	Mean	Std Deviation	Variance	Count
1	Dear Expert Panel Member, Thank you for returning the first round Delphi questionnaire. Your input makes us one step closer to the development of antidote stocking guidelines for Kuwait. You will now find the second (and final) round Delphi questionnaire which includes the responses of the whole expert panel towards the inclusion of each antidote into the Kuwaiti guidelines. In summary: Out of 47 antidotes, 41 were found to reach consensus (>75% agreement by all experts) 6 antidotes did not reach consensus (achieved 7 new antidotes were suggested to be added to the list. Your task now is: To reconsider your responses to all the antidotes that have reached consensus (and to re-assess them if you would like to change your response). To re-assess the six antidotes that did not reach consensus. To rate your agreement on the inclusion of 7 new antidotes to the national antidote guidelines. Please complete the Delphi questionnaire as fully as you can. Thank you for your continued participation in this study. Yours sincerely, Sara Al-Ansari Final year pharmacy student Email: sara.alansari@hsc.edu.kw Telephone: (+965) 65633350 Dalal Al-Taweel Director, Kuwait Medicines Information Centre (KUMIC), Faculty of Pharmacy, Kuwait University. Email: d.altaweel@hsc.edu.kw Telephone: (+965) 24636897	1.00	1.00	1.00	0.00	0.00	22

#	Field	Choice Count	
1	Agree (I have no conflict of interest to disclose)	100.00%	22
2	Disagree	0.00%	0
			22

Showing rows 1 - 3 of 3

Q1 - 1) Participant full name:

1) Participant full name:
Hussein Aldulaimi
Amira
Dr.Fatoumah Alabdulrazzaq
MOHAMMAD ALZAYED
essam nabeel alayoub
Huda Sadeq
Abdullah Alluhaidan
حاتم الفرا
Haider Ahmad
Abdulrahman ali Husain
Ali mohsen
Dr.Mohammad Hajeyah
Nadeem Abdulaziz Alduaij
Adnan Hajjiah
Ahmad Wajih Alqallaf
Abdullatif Aloumi
Selma Alqattan
Ahmad alshadad
Faisal Alghanem
Laila Shehab Ahmed
Mariam Mahmood
Abdulaziz Alburaidi

Q2 - 2) Re-assess the 6 antidotes that did not reach consensus.

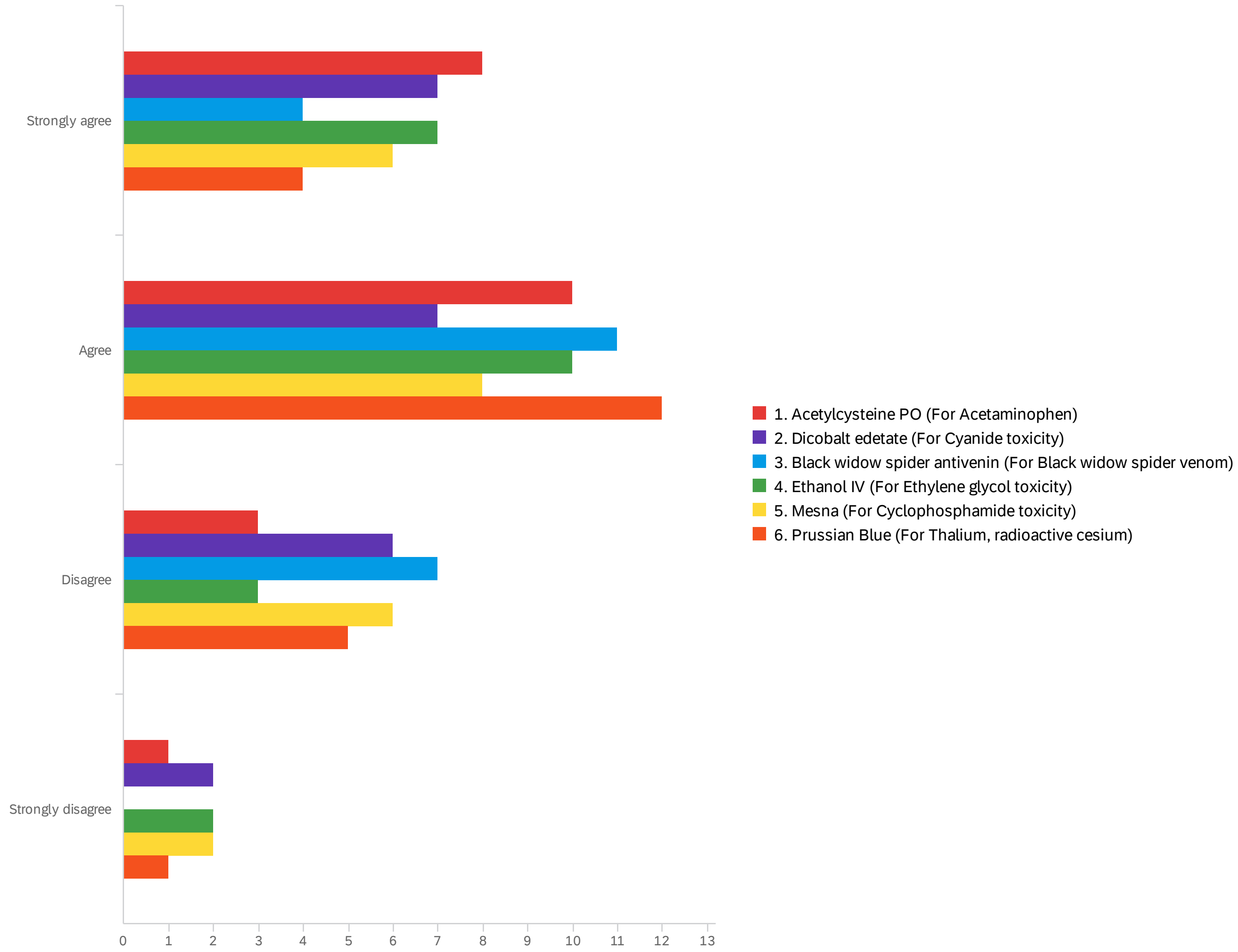

#	Field	Minimum	Maximum	Mean	Std Deviation	Variance	Count
1	1. Acetylcysteine PO (For Acetaminophen)	4.00	10.00	5.41	1.87	3.51	22
2	2. Dicobalt edetate (For Cyanide toxicity)	4.00	10.00	6.23	2.33	5.45	22
3	3. Black widow spider antivenin (For Black widow spider venom)	4.00	9.00	6.09	2.02	4.08	22
4	4. Ethanol IV (For Ethylene glycol toxicity)	4.00	10.00	5.68	2.08	4.31	22
5	5. Mesna (For Cyclophosphamide toxicity)	4.00	10.00	6.27	2.30	5.29	22
6	6. Prussian Blue (For Thalium, radioactive cesium)	4.00	10.00	5.95	2.01	4.04	22

#	Field	Strongly agree		Agree		Disagree		Strongly disagree		Total
1	1. Acetylcysteine PO (For Acetaminophen)	36.36%	8	45.45%	10	13.64%	3	4.55%	1	22
2	2. Dicobalt edetate (For Cyanide toxicity)	31.82%	7	31.82%	7	27.27%	6	9.09%	2	22
3	3. Black widow spider antivenin (For Black widow spider venom)	18.18%	4	50.00%	11	31.82%	7	0.00%	0	22
4	4. Ethanol IV (For Ethylene glycol toxicity)	31.82%	7	45.45%	10	13.64%	3	9.09%	2	22
5	5. Mesna (For Cyclophosphamide toxicity)	27.27%	6	36.36%	8	27.27%	6	9.09%	2	22
6	6. Prussian Blue (For Thalium, radioactive cesium)	18.18%	4	54.55%	12	22.73%	5	4.55%	1	22

Showing rows 1 - 6 of 6

Q6 - 3) Rate your level of agreement to the inclusion of the 7 antidotes below

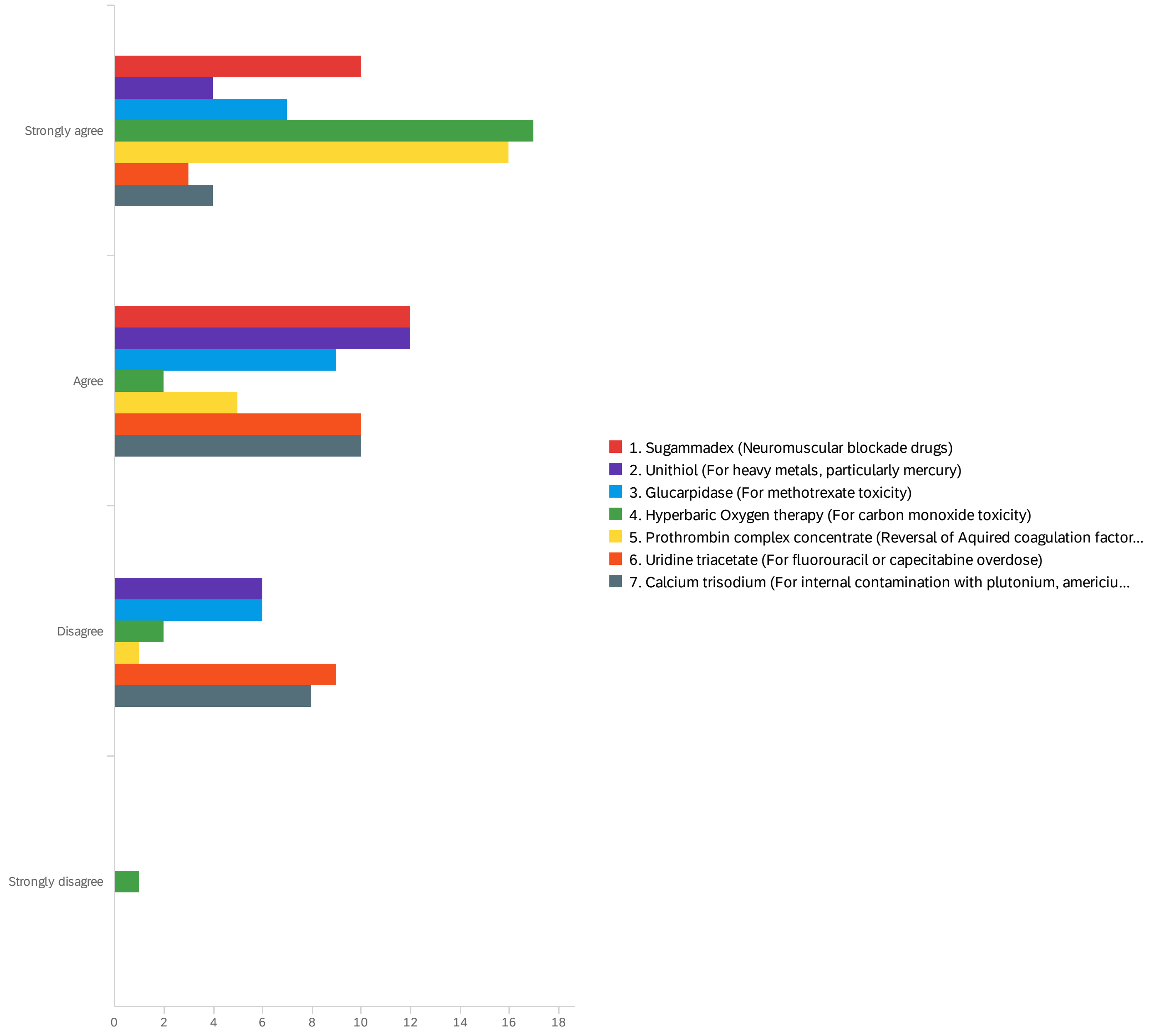

#	Field	Minimum	Maximum	Mean	Std Deviation	Variance	Count
1	1. Sugammadex (Neuromuscular blockade drugs)	4.00	5.00	4.55	0.50	0.25	22
2	2. Unithiol (For heavy metals, particularly mercury)	4.00	9.00	5.91	1.93	3.72	22
3	3. Glucarpidase (For methotrexate toxicity)	4.00	9.00	5.77	2.02	4.08	22
4	4. Hyperbaric Oxygen therapy (For carbon monoxide toxicity)	4.00	10.00	4.82	1.82	3.33	22

#	Field	Minimum	Maximum	Mean	Std Deviation	Variance	Count
5	5. Prothrombin complex concentrate (Reversal of Aquired coagulation factor deficiency induced by Vitamin K Antagonists)	4.00	9.00	4.45	1.08	1.16	22
6	6. Uridine triacetate (For fluorouracil or capecitabine overdose)	4.00	9.00	6.50	2.11	4.43	22
7	7. Calcium trisodium (For internal contamination with plutonium, americium, or curium)	4.00	9.00	6.27	2.09	4.38	22

#	Field	Strongly agree		Agree		Disagree		Strongly disagree		Total
1	1. Sugammadex (Neuromuscular blockade drugs)	45.45%	10	54.55%	12	0.00%	0	0.00%	0	22
2	2. Unithiol (For heavy metals, particularly mercury)	18.18%	4	54.55%	12	27.27%	6	0.00%	0	22
3	3. Glucarpidase (For methotrexate toxicity)	31.82%	7	40.91%	9	27.27%	6	0.00%	0	22
4	4. Hyperbaric Oxygen therapy (For carbon monoxide toxicity)	77.27%	17	9.09%	2	9.09%	2	4.55%	1	22
5	5. Prothrombin complex concentrate (Reversal of Aquired coagulation factor deficiency induced by Vitamin K Antagonists)	72.73%	16	22.73%	5	4.55%	1	0.00%	0	22
6	6. Uridine triacetate (For fluorouracil or capecitabine overdose)	13.64%	3	45.45%	10	40.91%	9	0.00%	0	22
7	7. Calcium trisodium (For internal contamination with plutonium, americium, or curium)	18.18%	4	45.45%	10	36.36%	8	0.00%	0	22

Showing rows 1 - 7 of 7

Q4 - 4) If you have any comments, you can add them below (OPTIONAL)

4) If you have any comments, you can add them below (OPTIONAL)
Dicobalt edetate: has little data to support it, only used in some parts of Europe. has a very poor side effect profile when used on patients that don't have cyanide toxicity but were given as a suspicion only. Plus, there are 2 much safer alternatives.
Hyperbaric oxygen while is the mainstay of treatment for CO poisoning, it is a large device and not a pharmaceutical agent.

Optional - Would you like to re-assess any of the 41 antidotes that already reached consensus?

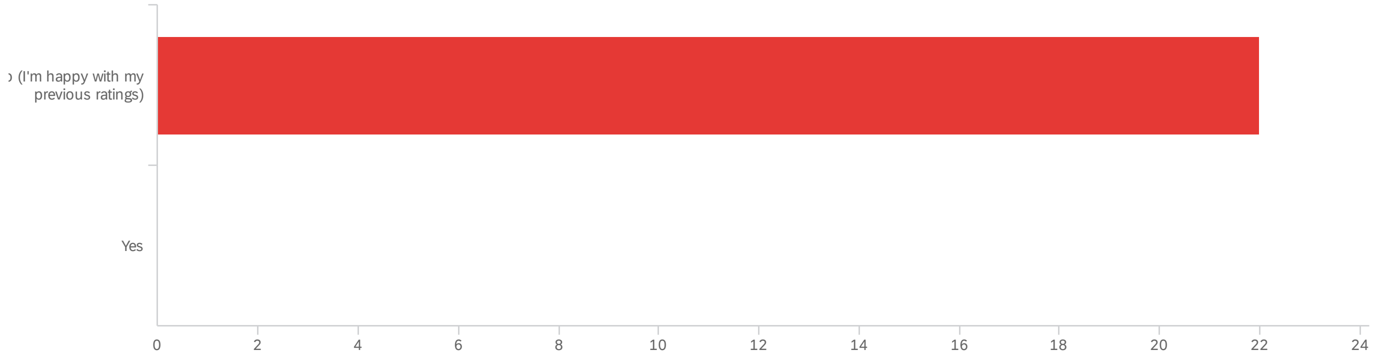

#	Field	Minimum	Maximum	Mean	Std Deviation	Variance	Count
1	Would you like to re-assess any of the 41 antidotes that already reached consensus?	1.00	1.00	1.00	0.00	0.00	22

#	Field	Choice Count	
1	No (I'm happy with my previous ratings)	100.00%	22
2	Yes	0.00%	0
			22

Showing rows 1 - 3 of 3

Optional - NOTE: THIS QUESTION IS OPTIONAL We will only consider the new change!

If your opinion of the other antidotes is the same as round one, leave them empty or click on the option "same opinion as round one". we will take the result of round one for these antidotes.

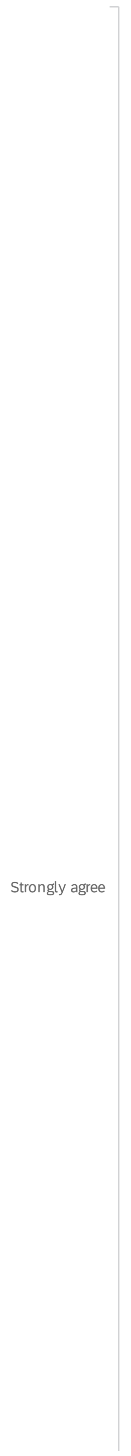

Agree

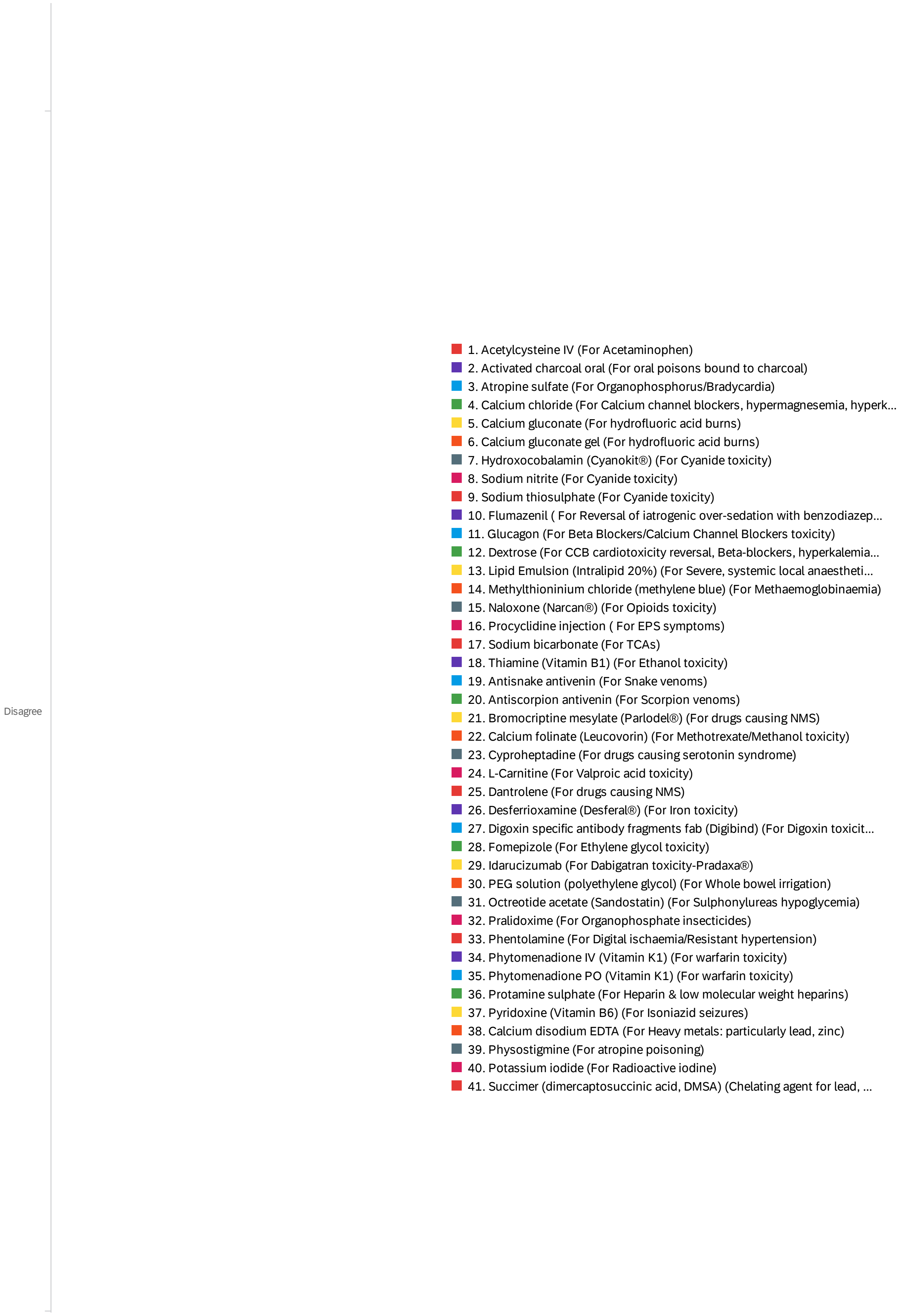
Disagree
1. Acetylcysteine IV (For Acetaminophen)
2. Activated charcoal oral (For oral poisons bound to charcoal)
3. Atropine sulfate (For Organophosphorus/Bradycardia)
4. Calcium chloride (For Calcium channel blockers, hypermagnesemia, hyperk...
5. Calcium gluconate (For hydrofluoric acid burns)
6. Calcium gluconate gel (For hydrofluoric acid burns)
7. Hydroxocobalamin (Cyanokit®) (For Cyanide toxicity)
8. Sodium nitrite (For Cyanide toxicity)
9. Sodium thiosulphate (For Cyanide toxicity)
10. Flumazenil (For Reversal of iatrogenic over-sedation with benzodiazep...
11. Glucagon (For Beta Blockers/Calcium Channel Blockers toxicity)
12. Dextrose (For CCB cardiotoxicity reversal, Beta-blockers, hyperkalemia...
13. Lipid Emulsion (Intralipid 20%) (For Severe, systemic local anaestheti...
14. Methylthioninium chloride (methylene blue) (For Methaemoglobinaemia)
15. Naloxone (Narcan®) (For Opioids toxicity)
16. Procyclidine injection (For EPS symptoms)
17. Sodium bicarbonate (For TCAs)
18. Thiamine (Vitamin B1) (For Ethanol toxicity)
19. Antisnake antivenin (For Snake venoms)
20. Antiscorpion antivenin (For Scorpion venoms)
21. Bromocriptine mesylate (Parlodel®) (For drugs causing NMS)
22. Calcium folinate (Leucovorin) (For Methotrexate/Methanol toxicity)
23. Cyproheptadine (For drugs causing serotonin syndrome)
24. L-Carnitine (For Valproic acid toxicity)
25. Dantrolene (For drugs causing NMS)
26. Desferrioxamine (Desferal®) (For Iron toxicity)
27. Digoxin specific antibody fragments fab (Digibind) (For Digoxin toxicit...
28. Fomepizole (For Ethylene glycol toxicity)
29. Idarucizumab (For Dabigatran toxicity-Pradaxa®)
30. PEG solution (polyethylene glycol) (For Whole bowel irrigation)
31. Octreotide acetate (Sandostatin) (For Sulphonylureas hypoglycemia)
32. Pralidoxime (For Organophosphate insecticides)
33. Phentolamine (For Digital ischaemia/Resistant hypertension)
34. Phytomenadione IV (Vitamin K1) (For warfarin toxicity)
35. Phytomenadione PO (Vitamin K1) (For warfarin toxicity)
36. Protamine sulphate (For Heparin & low molecular weight heparins)
37. Pyridoxine (Vitamin B6) (For Isoniazid seizures)
38. Calcium disodium EDTA (For Heavy metals: particularly lead, zinc)
39. Physostigmine (For atropine poisoning)
40. Potassium iodide (For Radioactive iodine)
41. Succimer (dimercaptosuccinic acid, DMSA) (Chelating agent for lead, ...

Strongly disagree

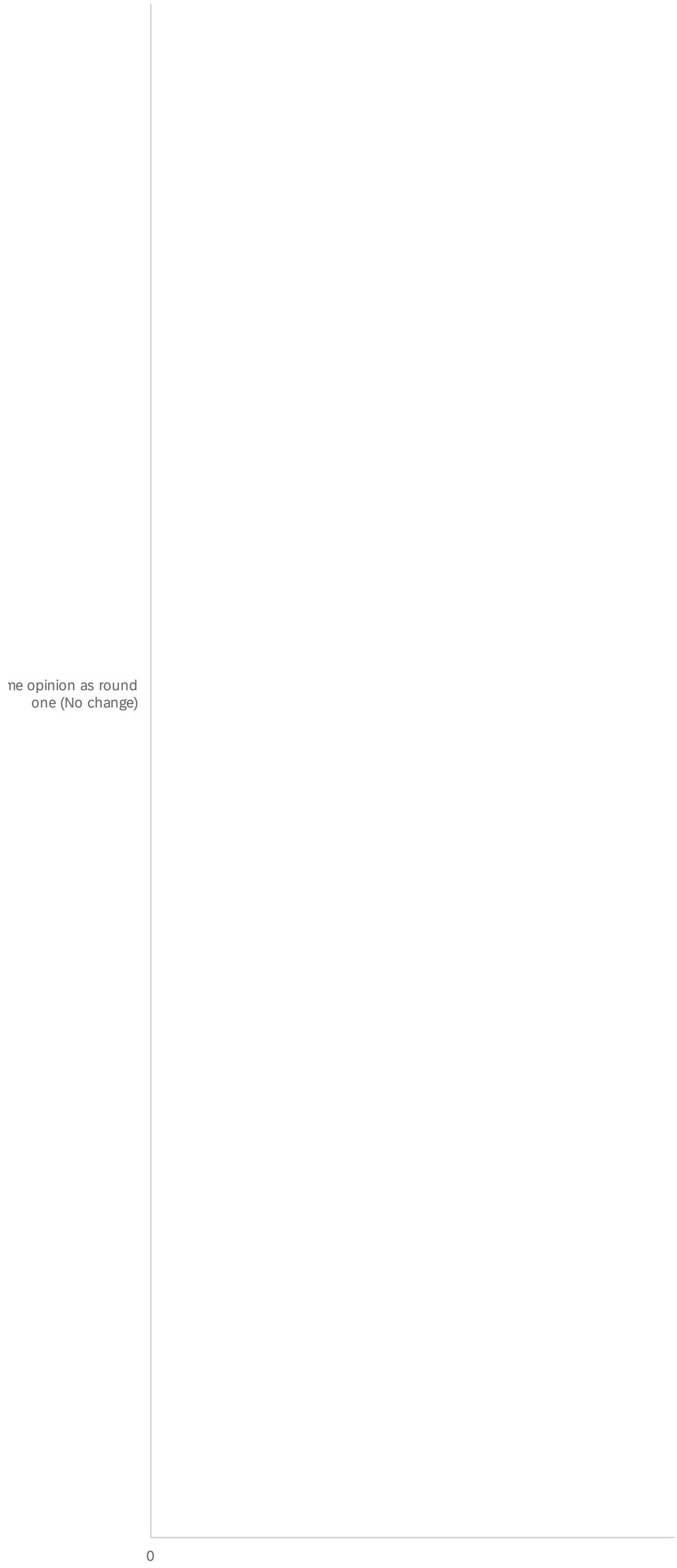

#	Field	Minimum	Maximum	Mean	Std Deviation	Variance	Count
1	1. Acetylcysteine IV (For Acetaminophen)	0.00	0.00	0.00	0.00	0.00	0

#	Field	Minimum	Maximum	Mean	Std Deviation	Variance	Count
2	2. Activated charcoal oral (For oral poisons bound to charcoal)	0.00	0.00	0.00	0.00	0.00	0
3	3. Atropine sulfate (For Organophosphorus/Bradycardia)	0.00	0.00	0.00	0.00	0.00	0
4	4. Calcium chloride (For Calcium channel blockers, hypermagnesemia, hyperkalemia)	0.00	0.00	0.00	0.00	0.00	0
5	5. Calcium gluconate (For hydrofluoric acid burns)	0.00	0.00	0.00	0.00	0.00	0
6	6. Calcium gluconate gel (For hydrofluoric acid burns)	0.00	0.00	0.00	0.00	0.00	0
7	7. Hydroxocobalamin (Cyanokit®) (For Cyanide toxicity)	0.00	0.00	0.00	0.00	0.00	0
8	8. Sodium nitrite (For Cyanide toxicity)	0.00	0.00	0.00	0.00	0.00	0
9	9. Sodium thiosulphate (For Cyanide toxicity)	0.00	0.00	0.00	0.00	0.00	0
10	10. Flumazenil (For Reversal of iatrogenic over-sedation with benzodiazepines)	0.00	0.00	0.00	0.00	0.00	0
11	11. Glucagon (For Beta Blockers/Calcium Channel Blockers toxicity)	0.00	0.00	0.00	0.00	0.00	0
12	12. Dextrose (For CCB cardiotoxicity reversal, Beta-blockers, hyperkalemia)	0.00	0.00	0.00	0.00	0.00	0
13	13. Lipid Emulsion (Intralipid 20%) (For Severe, systemic local anaesthetic toxicity)	0.00	0.00	0.00	0.00	0.00	0
14	14. Methylthioninium chloride (methylene blue) (For Methaemoglobinaemia)	0.00	0.00	0.00	0.00	0.00	0
15	15. Naloxone (Narcan®) (For Opioids toxicity)	0.00	0.00	0.00	0.00	0.00	0
16	16. Procyclidine injection (For EPS symptoms)	0.00	0.00	0.00	0.00	0.00	0
17	17. Sodium bicarbonate (For TCAs)	0.00	0.00	0.00	0.00	0.00	0
18	18. Thiamine (Vitamin B1) (For Ethanol toxicity)	0.00	0.00	0.00	0.00	0.00	0
19	19. Antisnake antivenin (For Snake venoms)	0.00	0.00	0.00	0.00	0.00	0
20	20. Antiscorpion antivenin (For Scorpion venoms)	0.00	0.00	0.00	0.00	0.00	0
21	21. Bromocriptine mesylate (Parlodel®) (For drugs causing NMS)	0.00	0.00	0.00	0.00	0.00	0
22	22. Calcium folinate (Leucovorin) (For Methotrexate/Methanol toxicity)	0.00	0.00	0.00	0.00	0.00	0
23	23. Cyproheptadine (For drugs causing serotonin syndrome)	0.00	0.00	0.00	0.00	0.00	0

#	Field	Minimum	Maximum	Mean	Std Deviation	Variance	Count
24	24. L-Carnitine (For Valproic acid toxicity)	0.00	0.00	0.00	0.00	0.00	0
25	25. Dantrolene (For drugs causing NMS)	0.00	0.00	0.00	0.00	0.00	0
26	26. Desferrioxamine (Desferal®) (For Iron toxicity)	0.00	0.00	0.00	0.00	0.00	0
27	27. Digoxin specific antibody fragments fab (Digibind) (For Digoxin toxicity)	0.00	0.00	0.00	0.00	0.00	0
28	28. Fomepizole (For Ethylene glycol toxicity)	0.00	0.00	0.00	0.00	0.00	0
29	29. Idarucizumab (For Dabigatran toxicity-Pradaxa®)	0.00	0.00	0.00	0.00	0.00	0
30	30. PEG solution (polyethylene glycol) (For Whole bowel irrigation)	0.00	0.00	0.00	0.00	0.00	0
31	31. Octreotide acetate (Sandostatin) (For Sulphonylureas hypoglycemia)	0.00	0.00	0.00	0.00	0.00	0
32	32. Pralidoxime (For Organophosphate insecticides)	0.00	0.00	0.00	0.00	0.00	0
33	33. Phentolamine (For Digital ischaemia/Resistant hypertension)	0.00	0.00	0.00	0.00	0.00	0
34	34. Phytomenadione IV (Vitamin K1) (For warfarin toxicity)	0.00	0.00	0.00	0.00	0.00	0
35	35. Phytomenadione PO (Vitamin K1) (For warfarin toxicity)	0.00	0.00	0.00	0.00	0.00	0
36	36. Protamine sulphate (For Heparin & low molecular weight heparins)	0.00	0.00	0.00	0.00	0.00	0
37	37. Pyridoxine (Vitamin B6) (For Isoniazid seizures)	0.00	0.00	0.00	0.00	0.00	0
38	38. Calcium disodium EDTA (For Heavy metals: particularly lead, zinc)	0.00	0.00	0.00	0.00	0.00	0
39	39. Physostigmine (For atropine poisoning)	0.00	0.00	0.00	0.00	0.00	0
40	40. Potassium iodide (For Radioactive iodine)	0.00	0.00	0.00	0.00	0.00	0
41	41. Succimer (dimercaptosuccinic acid, DMSA) (Chelating agent for lead, mercury)	0.00	0.00	0.00	0.00	0.00	0

#	Field	Strongly agree		Agree		Disagree		Strongly disagree		Same opinion as round one (No change)		Total
1	1. Acetylcysteine IV (For Acetaminophen)	0.00%	0	0.00%	0	0.00%	0	0.00%	0	0.00%	0	0

#	Field	Strongly agree		Agree		Disagree		Strongly disagree		Same opinion as round one (No change)		Total
2	2. Activated charcoal oral (For oral poisons bound to charcoal)	0.00%	0	0.00%	0	0.00%	0	0.00%	0	0.00%	0	0
3	3. Atropine sulfate (For Organophosphorus/Bradycardia)	0.00%	0	0.00%	0	0.00%	0	0.00%	0	0.00%	0	0
4	4. Calcium chloride (For Calcium channel blockers, hypermagnesemia, hyperkalemia)	0.00%	0	0.00%	0	0.00%	0	0.00%	0	0.00%	0	0
5	5. Calcium gluconate (For hydrofluoric acid burns)	0.00%	0	0.00%	0	0.00%	0	0.00%	0	0.00%	0	0
6	6. Calcium gluconate gel (For hydrofluoric acid burns)	0.00%	0	0.00%	0	0.00%	0	0.00%	0	0.00%	0	0
7	7. Hydroxocobalamin (Cyanokit®) (For Cyanide toxicity)	0.00%	0	0.00%	0	0.00%	0	0.00%	0	0.00%	0	0
8	8. Sodium nitrite (For Cyanide toxicity)	0.00%	0	0.00%	0	0.00%	0	0.00%	0	0.00%	0	0
9	9. Sodium thiosulphate (For Cyanide toxicity)	0.00%	0	0.00%	0	0.00%	0	0.00%	0	0.00%	0	0
10	10. Flumazenil (For Reversal of iatrogenic over-sedation with benzodiazepines)	0.00%	0	0.00%	0	0.00%	0	0.00%	0	0.00%	0	0
11	11. Glucagon (For Beta Blockers/Calcium Channel Blockers toxicity)	0.00%	0	0.00%	0	0.00%	0	0.00%	0	0.00%	0	0
12	12. Dextrose (For CCB cardiotoxicity reversal, Beta-blockers, hyperkalemia)	0.00%	0	0.00%	0	0.00%	0	0.00%	0	0.00%	0	0
13	13. Lipid Emulsion (Intralipid 20%) (For Severe, systemic local anaesthetic toxicity)	0.00%	0	0.00%	0	0.00%	0	0.00%	0	0.00%	0	0
14	14. Methylthioninium chloride (methylene blue) (For Methaemoglobinaemia)	0.00%	0	0.00%	0	0.00%	0	0.00%	0	0.00%	0	0
15	15. Naloxone (Narcan®) (For Opioids toxicity)	0.00%	0	0.00%	0	0.00%	0	0.00%	0	0.00%	0	0
16	16. Procyclidine injection (For EPS symptoms)	0.00%	0	0.00%	0	0.00%	0	0.00%	0	0.00%	0	0
17	17. Sodium bicarbonate (For TCAs)	0.00%	0	0.00%	0	0.00%	0	0.00%	0	0.00%	0	0
18	18. Thiamine (Vitamin B1) (For Ethanol toxicity)	0.00%	0	0.00%	0	0.00%	0	0.00%	0	0.00%	0	0
19	19. Antisnake antivenin (For Snake venoms)	0.00%	0	0.00%	0	0.00%	0	0.00%	0	0.00%	0	0
20	20. Antiscorpion antivenin (For Scorpion venoms)	0.00%	0	0.00%	0	0.00%	0	0.00%	0	0.00%	0	0
21	21. Bromocriptine mesylate (Parlodel®) (For drugs causing NMS)	0.00%	0	0.00%	0	0.00%	0	0.00%	0	0.00%	0	0
22	22. Calcium folinate (Leucovorin) (For Methotrexate/Methanol toxicity)	0.00%	0	0.00%	0	0.00%	0	0.00%	0	0.00%	0	0
23	23. Cyproheptadine (For drugs causing serotonin syndrome)	0.00%	0	0.00%	0	0.00%	0	0.00%	0	0.00%	0	0

#	Field	Strongly agree		Agree		Disagree		Strongly disagree		Same opinion as round one (No change)		Total
24	24. L-Carnitine (For Valproic acid toxicity)	0.00%	0	0.00%	0	0.00%	0	0.00%	0	0.00%	0	0
25	25. Dantrolene (For drugs causing NMS)	0.00%	0	0.00%	0	0.00%	0	0.00%	0	0.00%	0	0
26	26. Desferrioxamine (Desferal®) (For Iron toxicity)	0.00%	0	0.00%	0	0.00%	0	0.00%	0	0.00%	0	0
27	27. Digoxin specific antibody fragments fab (Digibind) (For Digoxin toxicity)	0.00%	0	0.00%	0	0.00%	0	0.00%	0	0.00%	0	0
28	28. Fomepizole (For Ethylene glycol toxicity)	0.00%	0	0.00%	0	0.00%	0	0.00%	0	0.00%	0	0
29	29. Idarucizumab (For Dabigatran toxicity-Pradaxa®)	0.00%	0	0.00%	0	0.00%	0	0.00%	0	0.00%	0	0
30	30. PEG solution (polyethylene glycol) (For Whole bowel irrigation)	0.00%	0	0.00%	0	0.00%	0	0.00%	0	0.00%	0	0
31	31. Octreotide acetate (Sandostatin) (For Sulphonylureas hypoglycemia)	0.00%	0	0.00%	0	0.00%	0	0.00%	0	0.00%	0	0
32	32. Pralidoxime (For Organophosphate insecticides)	0.00%	0	0.00%	0	0.00%	0	0.00%	0	0.00%	0	0
33	33. Phentolamine (For Digital ischaemia/Resistant hypertension)	0.00%	0	0.00%	0	0.00%	0	0.00%	0	0.00%	0	0
34	34. Phytomenadione IV (Vitamin K1) (For warfarin toxicity)	0.00%	0	0.00%	0	0.00%	0	0.00%	0	0.00%	0	0
35	35. Phytomenadione PO (Vitamin K1) (For warfarin toxicity)	0.00%	0	0.00%	0	0.00%	0	0.00%	0	0.00%	0	0
36	36. Protamine sulphate (For Heparin & low molecular weight heparins)	0.00%	0	0.00%	0	0.00%	0	0.00%	0	0.00%	0	0
37	37. Pyridoxine (Vitamin B6) (For Isoniazid seizures)	0.00%	0	0.00%	0	0.00%	0	0.00%	0	0.00%	0	0
38	38. Calcium disodium EDTA (For Heavy metals: particularly lead, zinc)	0.00%	0	0.00%	0	0.00%	0	0.00%	0	0.00%	0	0
39	39. Physostigmine (For atropine poisoning)	0.00%	0	0.00%	0	0.00%	0	0.00%	0	0.00%	0	0
40	40. Potassium iodide (For Radioactive iodine)	0.00%	0	0.00%	0	0.00%	0	0.00%	0	0.00%	0	0
41	41. Succimer (dimercaptosuccinic acid, DMSA) (Chelating agent for lead, mercury)	0.00%	0	0.00%	0	0.00%	0	0.00%	0	0.00%	0	0

Showing rows 1 - 41 of 41

End of Report
