# Supplementary material for: Expert consensus for a national essential antidote list: E-Delphi method
Source: PLoS One. 2022 Jun 16;17(6):e0269456. doi: 10.1371/journal.pone.0269456 (PMC9202922; doi:10.1371/journal.pone.0269456)
Supplement: S3 File — (PDF) [file pone.0269456.s003.pdf]

Default Report

Round 3

December 2, 2020 10:28 AM EAT

Intro - Welcome to the third and FINAL round of the Delphi study to develop the Kuwait National Antidote guidelines. Thank you for your support in completing Round 1 and 2. We are now one step closer to achieving our goal in development of antidote sticking guidelines for Kuwait. As a reminder, Round 1: • Included 47 antidotes • the expert panel agreed on including 41 into the Kuwait National Antidote Guidlines. • 7 new antidotes were suggested by the expert panel. Round 2: • The expert panel re-assessed the 6 antidotes that did not reach consensus in Round 1 • Also, they assessed the 7 newly suggested antidotes. Result: six antidotes were removed as they did not reach consensus (for the second time) Round 3 (current round): • Experts are asked to re-assess their agreement level for the 7 "newly" suggested antidotes. Yours sincerely, Sara Al-Ansari Final year pharmacy student Email: sara.alansari@hsc.edu.kw Telephone: (+965) 65633350 Dalal Al-Taweel Director, Kuwait Medicines Information Centre (KUMIC), Faculty of Pharmacy, Kuwait University. Email: d.altaweel@hsc.edu.kw Telephone: (+965) 24636897

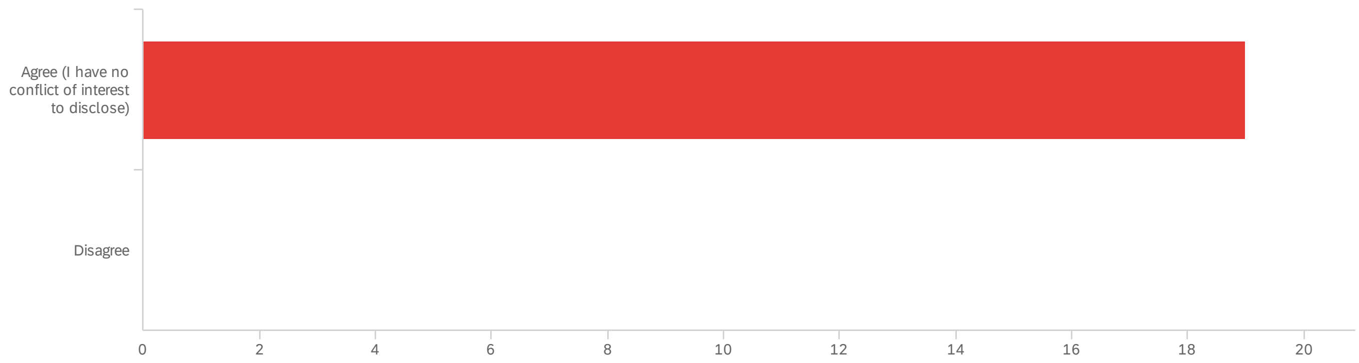

Data source misconfigured for this visualization.

Data source misconfigured for this visualization.

Q1 - 1) Participant full name:

WIDGET_ERROR.MISCONFIGURED

Q2 - 2) Re-assess the 5 antidotes that did not reach consensus during round 2

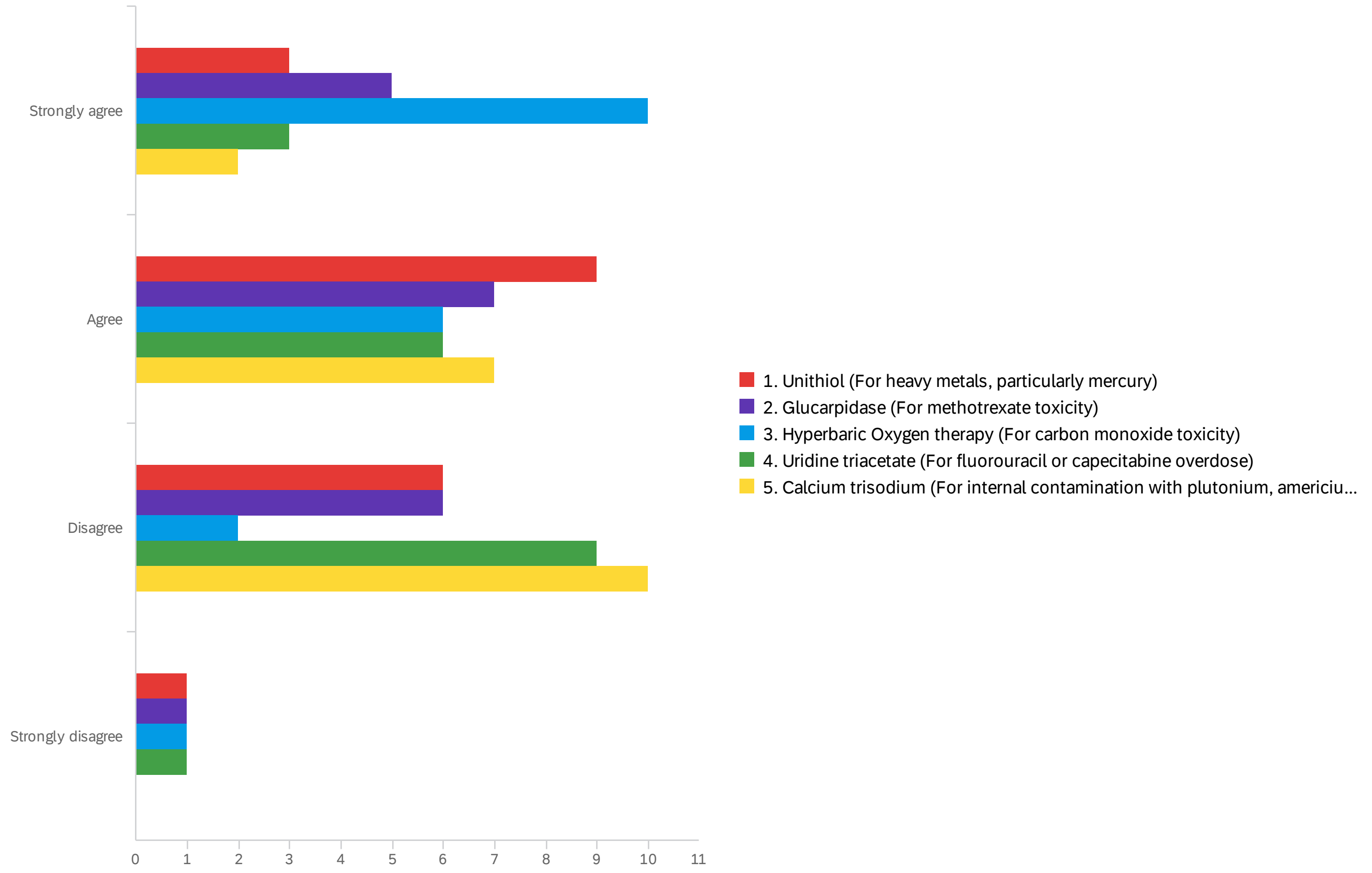

#	Field	Minimum	Maximum	Mean	Std Deviation	Variance	Count
1	1. Unithiol (For heavy metals, particularly mercury)	4.00	10.00	6.37	2.16	4.65	19
2	2. Glucarpidase (For methotrexate toxicity)	4.00	10.00	6.26	2.24	5.04	19
3	3. Hyperbaric Oxygen therapy (For carbon monoxide toxicity)	4.00	10.00	5.16	1.87	3.50	19
4	4. Uridine triacetate (For fluorouracil or capecitabine overdose)	4.00	10.00	7.00	2.25	5.05	19
5	5. Calcium trisodium (For internal contamination with plutonium, americium, or curium)	4.00	9.00	7.00	2.13	4.53	19

#	Field	Strongly agree		Agree		Disagree		Strongly disagree		Total
1	1. Unithiol (For heavy metals, particularly mercury)	15.79%	3	47.37%	9	31.58%	6	5.26%	1	19
2	2. Glucarpidase (For methotrexate toxicity)	26.32%	5	36.84%	7	31.58%	6	5.26%	1	19

#	Field	Strongly agree		Agree		Disagree		Strongly disagree		Total
3	3. Hyperbaric Oxygen therapy (For carbon monoxide toxicity)	52.63%	10	31.58%	6	10.53%	2	5.26%	1	19
4	4. Uridine triacetate (For fluorouracil or capecitabine overdose)	15.79%	3	31.58%	6	47.37%	9	5.26%	1	19
5	5. Calcium trisodium (For internal contamination with plutonium, americium, or curium)	10.53%	2	36.84%	7	52.63%	10	0.00%	0	19

Showing rows 1 - 5 of 5

Q3 - 3) Comments (OPTIONAL)

3) Comments (OPTIONAL)
No so common
Evidence for HBOT in CO is excessively weak. See Cochrane review 2011 (not recommended) and NHS policy 2018 (not recommended)
Suggest reconsideration of oral nAcetylcystine

Optional - Would you like to re-assess any of the 2 antidotes that already reached consensus during Round 2? (Sugammadex and prothrombin complex concentrate)

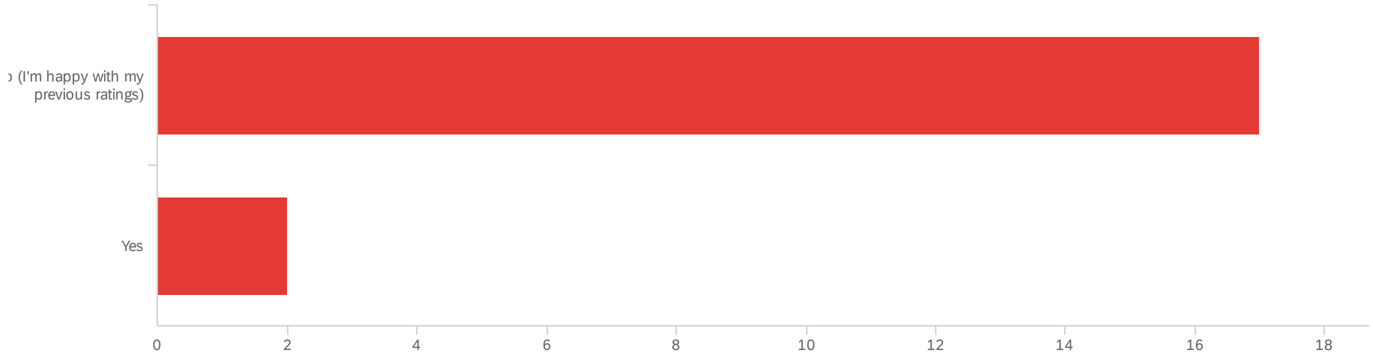

#	Field	Minimum	Maximum	Mean	Std Deviation	Variance	Count
1	Would you like to re-assess any of the 2 antidotes that already reached consensus during Round 2? (Sugammadex and prothrombin complex concentrate)	1.00	2.00	1.11	0.31	0.09	19

#	Field	Choice Count	
1	No (I'm happy with my previous ratings)	89.47%	17
2	Yes	10.53%	2
			19

Showing rows 1 - 3 of 3

Optional - NOTE: THIS QUESTION IS OPTIONAL We will only consider the new change!

If your opinion of the other antidotes is the same as round one, leave them empty or click on the option "same opinion as round one". we will take the result of round one for these antidotes.

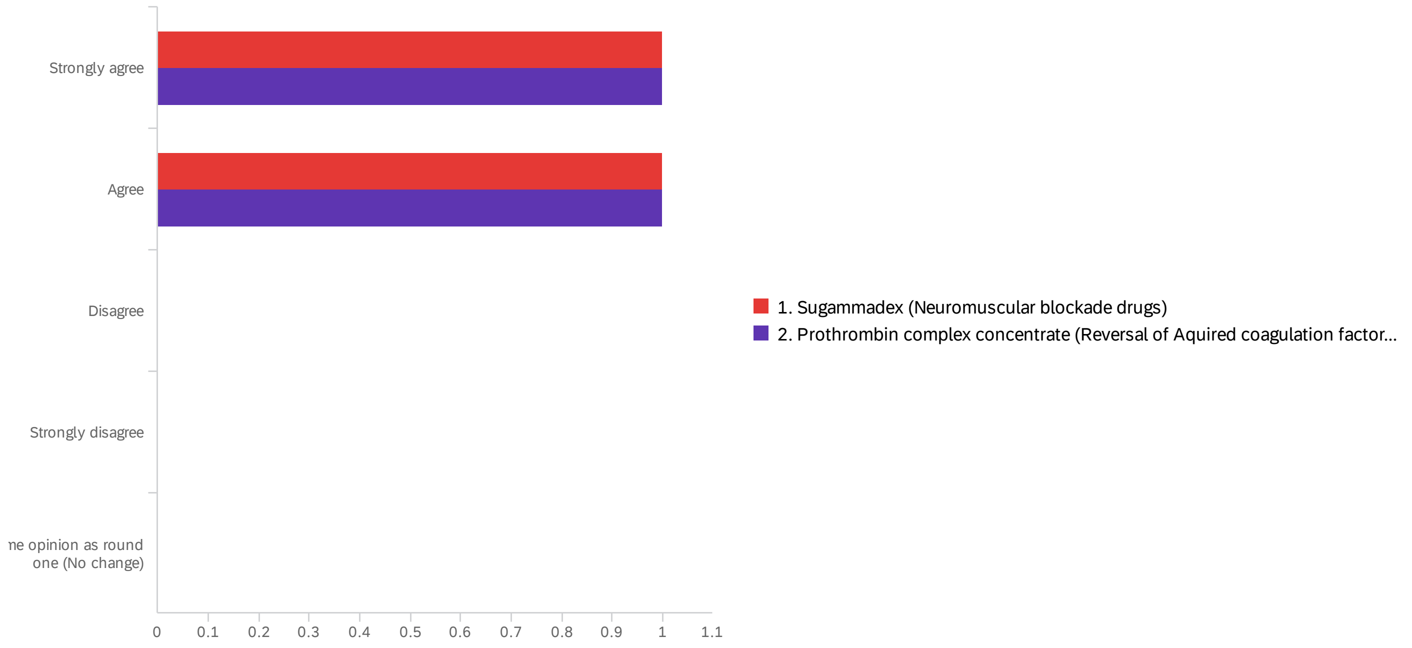

#	Field	Minimum	Maximum	Mean	Std Deviation	Variance	Count
1	1. Sugammadex (Neuromuscular blockade drugs)	26.00	27.00	26.50	0.50	0.25	2
2	2. Prothrombin complex concentrate (Reversal of Aquired coagulation factor deficiency induced by Vitamin K Antagonists)	26.00	27.00	26.50	0.50	0.25	2

#	Field	Strongly agree	Agree	Disagree	Strongly disagree	Same opinion as round one (No change)	Total
1	1. Sugammadex (Neuromuscular blockade drugs)	50.00% 1	50.00% 1	0.00% 0	0.00% 0	0.00% 0	2
2	2. Prothrombin complex concentrate (Reversal of Aquired coagulation factor deficiency induced by Vitamin K Antagonists)	50.00% 1	50.00% 1	0.00% 0	0.00% 0	0.00% 0	2

Showing rows 1 - 2 of 2

End of Report
